# BH3-mimetics or DNA-damaging agents in combination with RG7388 overcome p53 mutation-induced resistance to MDM2 inhibition

**DOI:** 10.1007/s10495-024-02014-8

**Published:** 2024-09-02

**Authors:** N. V. Pervushin, D. K. Nilov, S. V. Pushkarev, V. O. Shipunova, A. S. Badlaeva, M. A. Yapryntseva, D. V. Kopytova, B. Zhivotovsky, G. S. Kopeina

**Affiliations:** 1 Engelhardt Institute of Molecular Biology, Russian Academy of Sciences, Moscow, 119991 Russia; 2 Faculty of Medicine, MV Lomonosov Moscow State University, Moscow, 119991 Russia; 3 Belozersky Institute of Physicochemical Biology, Lomonosov Moscow State University, Moscow, 119991 Russia; 4 Faculty of Bioengineering and Bioinformatics, Lomonosov Moscow State University, Moscow, 119234 Russia; 5 Shemyakin-Ovchinnikov Institute of Bioorganic Chemistry, Russian Academy of Sciences, Moscow, 117997 Russia; 6 Moscow Center for Advanced Studies, Moscow, 123592 Russia; 7 Kulakov National Medical Research Center for Obstetrics, Gynecology and Perinatology, Russian Ministry of Health, Moscow, 117513 Russia; 8 Center for Precision Genome Editing and Genetic Technologies for Biomedicine, Engelhardt Institute of Molecular Biology, Russian Academy of Sciences, Moscow, 119991 Russia; 9 Division of Toxicology, Institute of Environmental Medicine, Karolinska Institutet, Box 210, 17177 Stockholm, Sweden

**Keywords:** MDM2, p53 mutation, Drug resistance, BH3-mimetics, RG7388

## Abstract

The development of drug resistance reduces the efficacy of cancer therapy. Tumor cells can acquire resistance to MDM2 inhibitors, which are currently under clinical evaluation. We generated RG7388-resistant neuroblastoma cells, which became more proliferative and metabolically active and were less sensitive to DNA-damaging agents in vitro and in vivo, compared with wild-type cells. The resistance was associated with a mutation of the p53 protein (His193Arg). This mutation abated its transcriptional activity via destabilization of the tetrameric p53-DNA complex and was observed in many cancer types. Finally, we found that Cisplatin and various BH3-mimetics could enhance RG7388-mediated apoptosis in RG7388-resistant neuroblastoma cells, thereby partially overcoming resistance to MDM2 inhibition.

**Supplementary Information:**

The online version contains supplementary material available at 10.1007/s10495-024-02014-8.

## Introduction

The p53 protein, encoded by the *TP53* gene, is a transcription factor that plays an essential role in maintaining cell genomic stability under normal and stressful conditions. This multidomain protein acts as the “guardian of the genome” and prevents the proliferation of cells that have undergone DNA damage. The p53 protein is also involved in the regulation of apoptosis and other types of programmed cell death (PCD), senescence, the cell cycle, and metabolism [1–4]. Importantly, the *TP53* gene is prone to mutations that lead to a loss of protein function. About 50% of human cancers express various mutations in the *TP53* gene. Tumors bearing *TP53* mutations are characterized by enhanced cell proliferation and poor prognosis in therapy [5].

The key regulator of p53 is the murine double minute 2 (MDM2) protein [6]. Under normal cellular conditions, this E3 ubiquitin ligase suppresses p53 levels via several mechanisms. At first, MDM2 can bind to p53 and target it for ubiquitination and subsequent proteasome degradation. Next, MDM2 leads to the nuclear export of p53 and prevents its activation at the transcriptional level. Moreover, p53 accumulation enhances MDM2 expression [6]. Additionally, murine double minute X (MDMX), a homolog of MDM2, also contributes to the regulation of p53 [7, 8].

Therefore, MDM2 inhibition represents a prospective therapeutic approach for tumors bearing wild-type (Wt) p53. Its accumulation leads to cell cycle arrest and PCD activation by inducing upregulation of p53-dependent proteins (e.g., p21, Bax, Puma) [9]. Back in 2004, the first class of small molecule inhibitors of MDM2, the members of the nutlin family were introduced and were shown to increase the level of p53 protein effectively [10]. Over the past two decades, many studies have been focused on the further development of these drugs [11–15]. Nowadays, several MDM2 inhibitors (RG7388, AMG-232, APG-115 and others) are being evaluated in clinical trials [12, 13].

Initially, it was suggested that tumor cells are less prone to acquiring resistance to MDM2 antagonists than to chemotherapeutic agents due to the non-genotoxic action of the former. Unfortunately, the problem of acquired drug resistance has also become associated with MDM2 inhibition [16]. Therefore, we decided to investigate the mechanisms underlying this type of resistance in SH-SY5Y neuroblastoma cells. This disease is one of the most common childhood tumors [17]. Importantly, *TP53* mutations are not typical of neuroblastoma tumors (about 1–2%). Furthermore, MDM2 inhibitors were found to demonstrate high efficacy in this type of cancer [18]. RG7388 was able to induce cell death and inhibit tumor growth in p53 Wt neuroblastoma cell lines, including SH-SY5Y [19].

Herein, the pulse-selection approach to obtaining resistant cells was used. This strategy represents a short drug exposure of chemotherapeutics to cancer cells at lower concentrations followed by a recovery period. This clinically relevant approach is similar to patients’ chemotherapy cycles [20]. Next, the biological properties of resistant cells and their sensitivity to chemotherapeutic drugs in vitro and in vivo were examined.

Notably, MDM2 inhibitors can trigger cell death in a restricted subset of cells containing the p53 Wt protein. Therefore, to improve anticancer activity, these inhibitors have been actively investigated in combination with various agents, including both chemotherapeutics and targeted therapies [12, 13, 21]. For instance, BH3-mimetics, small molecule inhibitors, disrupt protein complexes between pro- and antiapoptotic proteins of the Bcl-2 family. ABT-199/Venetoclax (a selective Bcl-2 inhibitor), being the first FDA-approved BH3-mimetic, as well as other drugs, are tested in anticancer trials [22–24]. Notably, ABT-199/Venetoclax was found to enhance RG7388-mediated apoptosis in neuroblastoma cells in vitro and in vivo, and now this combination is under clinical investigation [13, 25]. Here, the substitution His193Arg in p53 was observed to be associated with functional dysfunction of p53 and acquired resistance to MDM2 inhibition. The analysis of the strategies for overcoming this type of resistance using DNA damaging agents (Doxorubicin and Cisplatin) and BH3-mimetics revealed that the appearance of this mutation plays a vital role in the response of cancer cells to antitumor treatment.

## Materials and methods

### Cell culture

The neuroblastoma cell lines SH-SY5Y and SK-N-SH were supplied by the Department of Toxicology, Karolinska Institutet (Stockholm, Sweden) and were grown in a CO_2_ incubator (5% CO_2_) at + 37 °C. Cells were routinely checked for mycoplasma using the MycoReport kit (Evrogen, Moscow, Russia), analyzed by the STR approach, and re-cultured every 4 days in RPMI1640 medium (Gibco, Waltham, MA, USA) containing 10% fetal bovine serum (Gibco), a mixture of antibiotics and antimycotics (Gibco), and 1 mM sodium pyruvate (PanEco, Moscow, Russia).

### Reagents and experimental procedures

Cells in the logarithmic growth phase were utilized for the experiments. The culture medium was replaced each time before the addition of drugs. RG7388 (Idasanutlin) was purchased from Roche (Basel, Switzerland), S63845 was from Active Biochem (Hong Kong, China), ABT-199/Venetoclax and A1331852 were from Selleckchem (Houston, TX, USA), Cisplatin was from Teva (Petah Tikva, Israel), Doxorubicin hydrochloride (Doxorubicin) was from ABCR GmbH (Karlsruhe, Germany), and Zosuquidar hydrochloride (Zosuquidar) was from Sigma (Saint Louis, MO, USA).

### MTS assay

Briefly, SH-SY5Y cells (10^4^) were plated in 96-well plates (Nunc, Denmark). Then, fresh culture medium containing different concentrations of RG7388, Cisplatin, and Doxorubicin ranging from 0.01 to 100 µM were added to the cells. After treatment (for 24 h) MTS (CellTiter 96 AQueous One Solution Cell Proliferation Assay, Promega, Madison, WI, USA) reagent (20 µL) was added to each well. After incubation with MTS (3 h at + 37 °C), the spectrophotometric absorbance of the samples was measured at 490 nm using a VarioSkan Flash microplate reader (Thermo Fisher Scientific, Waltham, MA, USA). Half-maximal inhibitory concentrations (IC50) were calculated based on log values of technical duplicates and expressed as a mean and standard deviation (SD) (*n* = 4) using GraphPad Prism version 6 software (GraphPad Software, Inc., La Jolla, CA, USA).

### SubG1 test

The SubG1 test was performed as previously described [26]. Briefly, after detachment, fixation (70% ethanol), and washing, cells were stained with propidium iodide (Sigma, 20 µg/mL) and then analyzed with the BD FACSCanto II cell analyzer (BD Biosciences, San Jose, CA, USA). Data processing was performed with BD FACSDiva software 7.0 (BD Biosciences).

### Western blot (WB) analysis

Cells were collected, centrifuged (800 *g*, 5 min, + 4 °C), and washed with ice-cold PBS. The cell pellet was lysed in RIPA buffer (25 mM Tris-HCl (pH 7.4), 0.1% SDS, 150 mM NaCl, 1% NP-40, 0.5% sodium deoxycholate, and Halt protease inhibitor (Thermo Fisher Scientific) and phosphatase inhibitor cocktails (Sigma)) for 20 min on ice. After centrifugation (16,000 *g*, 20 min, + 4 °C), the supernatant was collected for protein determination with the Pierce BCA Protein Assay Kit (Thermo Fisher Scientific) and preparation of samples for western blot (WB) analysis, as previously described [27].

The following primary antibodies were used: anti-poly (ADP-ribose)-polymerase (PARP, ab137653) (Abcam, Cambridge, UK), anti-Bcl-2 (sc-509) (Santa Cruz Biotechnology, Dallas, TX, USA), anti-p53 (P6874-200UL, Sigma, Saint Louis, MO, USA), anti-PARP cleaved (#5625), anti-caspase-3 (full and cleaved) (#9662), anti-Mcl-1 (#5453), anti-Bax (#2772), anti-Puma (#4976), anti-Bcl-xL (#2764), anti-MDM2 (#86934), anti-p21 (#2946), anti-MDR1 (#12683), anti-glyceraldehyde 3-phosphate dehydrogenase (#2118) (all from Cell Signaling, Danvers, MA, USA). Anti-rabbit or anti-mouse IgG, conjugated with horseradish peroxidase (Jackson ImmunoResearch 111-035-144 (Ely, United Kingdom) and Jackson ImmunoResearch 515-035-062, respectively) were used as secondary antibodies. GAPDH was used as a loading control. Data processing and densitometric analysis of WB images were performed using Image Lab software.

### Quantitative reverse transcription PCR (RT-qPCR)

Total RNA was obtained from the cell pellet using TRIzol reagent (Thermo Fisher Scientific), and reverse transcription was performed using the MMLV RT kit (Evrogen) following the manufacturer’s instructions. Then, the qPCRmix-HS SYBR (Evrogen) and specific primers (Table S1) were added to cDNA samples, and mRNA expression was measured using the CFX96 Real-Time PCR Detection System (Bio-Rad). Transcript abundance was analyzed using Pfaffl’s method [28]. mRNA levels were normalized to the levels of TATA-binding protein (TBP) mRNA. The statistical analysis was conducted with GraphPad Prism version 6 software (GraphPad Software) using a two-tailed Student’s t-test with Welch’s correction (*n* = 3).

### Next-generation sequencing (NGS)

Genomic DNA isolated from wild-type SH-SY5Y (SH-SY5Y Wt) and SH-SY5Y resistant to RG7388 cells (SH-SY5Y Res) was sonicated to an average size of 300 nucleotides. According to the methodological recommendations, the library was prepared using the NEBNext^®^ Ultra^TM^ II DNA Library Prep Kit for Illumina (NEB, Ipswich, MA, USA). Then, it was used for hybridization with probes from the SureSelect Focused Exome kit (Agilent, Santa Clara, CA, USA), which includes probes specific to the coding regions of *TP53* and *MDM2*. Sequencing was performed on a HiSeq1500 instrument (Illumina, San Diego, CA, USA) to achieve an average coverage of 100 ×. The obtained sequences were aligned to the human genome version of Hg18, and mutations were searched manually.

### Clonogenic assay

Cells (10^3^ per well) were seeded in triplicate in 6-well plates (Nunc). After cultivation (10–14 days), cells were washed with PBS (twice), fixed with 4% paraformaldehyde solution (in PBS), and stained with crystal violet (0.5% in aqueous solution). The plates were imaged with the ChemiDoc XRS + System (Bio-Rad, Hercules, CA, USA) and analyzed using ImageJ software (version 1.53t).

### Metabolic assays

Cells were plated in a 96-well Seahorse microplate in RPMI1640 medium. After reaching 80% confluence, cells were washed and incubated in assay medium (RPMI1640 without FBS, phenol red, glycose, and sodium pyruvate supplemented with glutamine (2 mM) for glycolysis evaluation) for 1–3 h at + 37 °C. The same medium supplemented with sodium pyruvate (1 mM) and glucose (10 mM) was used for respiration evaluation. The metabolic assays were performed in real-time using the Seahorse XF Extracellular Flux Analyzer (Agilent, Santa Clara, CA, USA). For the respiration test (first three measurements) oligomycin (1 µM), carbonyl cyanide m-chlorophenyl hydrazone (CCCP, 1 µM), and rotenone/antimycin A1 (1 µM) were added to cells. For glycolysis evaluation D-Glucose (10 mM), oligomycin (1 µM), and 2-deoxyglucose (50 mM) were added to the wells. The data were normalized to the protein content in each well.

### Molecular modeling in silico

The molecular models of Wt and His193Arg mutant forms of p53 were constructed based on the 2ocj crystal structure (core DNA-binding domain) [29]. The N- and C-terminal ends of the protein were capped with ACE (acetyl) and NME (*N*-methylamide) groups using PyMOL 2.5 (Schrödinger, LLC; https://pymol.org). The imidazole ring of His214 located near the His193 residue was flipped to restore the local hydrogen bond network observed in other p53 structures. The His193Arg mutant was generated with Swiss-PdbViewer 4.1.0 by replacing the side chain of the 193 residue [30]. Then, the Wt and mutant structures were processed in the *tleap* program (AmberTools20 package) [31]. Hydrogen atoms were added considering the ionization of amino acid residues. The Wt His193 and His214 residues were modeled in the HIE form (with protonated N^ε2^ atom); pKa-ANI was used to predict the p*K*_a_ values of histidines [32]. The structures were placed in truncated octahedral TIP3P water boxes (the minimum distance to the box edge was 14 Å), and Na^+^ and Cl^–^ ions were added using the SPLIT method to achieve a salt concentration of 150 mM [33]. The protein was described with the *ff14SB* force field [34]; the Joung-Cheatham ion parameters [35] were used for Na^+^ and Cl^−^, and the Li-Merz parameters [36] were used for Zn^2+^ in the p53 zinc-binding motif.

The *pmemd* and *pmemd.cuda* programs (AMBER20 package) were used for energy minimization and molecular dynamics (MD) simulations, respectively [31, 37–39]. At the first stage of energy minimization (2500 steepest descent steps + 2500 conjugate gradient steps), the protein coordinates were kept fixed by positional restraints of 2 kcal/(mol Å^2^) on heavy atoms (Table S2). The second stage of minimization (5000 steepest descent steps + 5000 conjugate gradient steps) was conducted without any restraints. The obtained systems were then heated from 0 to 300 K using positional restraints of 1 kcal/(mol Å^2^) on the protein atoms (250 ps, constant volume) and equilibrated at 300 K (750 ps, constant pressure); three replicates run with randomized initial atomic velocities were performed for both Wt and mutant structures (Figure S1A). The SHAKE algorithm was used, allowing a time step of 0.002 ps. The temperature and pressure were controlled using the Langevin and Berendsen algorithms.

Starting from the equilibrated structures, conventional MD (cMD) simulations and Gaussian accelerated MD (GaMD) simulations were conducted at 300 K (constant pressure). The pressure was controlled using the Monte Carlo barostat. In GaMD simulations, preliminary 100 ns simulations were performed to adjust the molecular systems after switching to the GaMD potential and yield the required boost parameters (Figure S1A, Table S3). MD trajectories were analyzed using cpptraj 5.1.0 [40] and VMD 1.9 [41].

### In vivo tumor model

Female NSG (NOD/SCID/IL2rγnull) mice (weight = 22–25 g) were purchased and maintained at the Pushchino Animal Facility (Pushchino branch of the Shemyakin-Ovchinnikov Institute of Bioorganic Chemistry RAS, Institute, Pushchino, Russia). All procedures were approved by the Institutional Animal Care and Use Committee (IACUC) at this Institute (protocol #375/2023).

After reaching 90% confluence, SH-SY5Y Wt and SH-SY5Y Res (3 × 10^6^) cells were injected subcutaneously in a serum-free medium with 30% Matrigel (Corning) into the right flank of animals to create solid xenograft tumors. When the tumor volume reached ~ 50 mm^3^ (5 days after implantation), mice were randomly divided into four groups (*n* = 5 per group: 1—SH-SY5Y Wt «Control», 2—SH-SY5Y Wt «Doxorubicin», 3—SH-SY5Y Res to RG7388 «Control», 4—SH-SY5Y Res to RG7388 «Doxorubicin»). Then, mice from the «Doxorubicin» groups were injected intravenously with Doxorubicin (3 mg/kg/day), and mice from the «control» groups with saline solution (0.9% sodium chloride) three times (5–7 days after implantation). Tumor growth dynamics were monitored using a caliper by measuring the longitudinal and transverse dimensions, and the tumor volume was calculated as V = width^2^ × length/2. Mice were euthanized on day 12 after implantation.

### Histological analysis of tumors

Additional animals (*n* = 10) were recruited into two extra «control» groups (4 mice—SH-SY5Y Wt, 6—SH-SY5Y Res to RG7388 cells). Tumors were grown under the same conditions for 14 days. After mice were euthanized, xenograft tumors were resected and preserved in 10% neutral buffered formalin solution. FFPE (formalin-fixed paraffin-embedded) tissue samples were stained with hematoxylin and eosin. Immunohistochemical staining was performed on a Ventana BenchMark XT stainer (Ventana Medical-Systems, Oro Valley, AZ, USA) using Ki-67 primary antibody (Abcam, ab15580, dilution 1/100) and DAB Universal ultraView (Ventana Medical-Systems, Oro Valley, AZ, USA). The abovementioned antibody was incubated at + 37 °C for 16 min.

The M/A ratio (mitotic index to apoptotic index) was defined as the ratio of the number of mitotic figures to the number of apoptotic cells and estimated in 10 random high-power fields (10 HPF, ×400) of hematoxylin and eosin staining tumor slides. The Ki-67 expression rate was determined by counting the number of Ki-67-positive cells/500 or more tumor cells within a microscopic field.

### Data Processing and statistical analysis

The statistical analysis and data plotting were performed using GraphPad Prism version 6 software (GraphPad Software). The Mann–Whitney U-test was used to examine significant differences after four independent repeats unless stated otherwise in the figure legend: p-values lower than 0.05 were considered statistically significant; *ns *non-significant difference. Data are presented as mean ± standard deviation (SD).

## Results

### Development of acquired resistance to RG7388 (Idasanutlin) in neuroblastoma cells

**Fig. 1:**
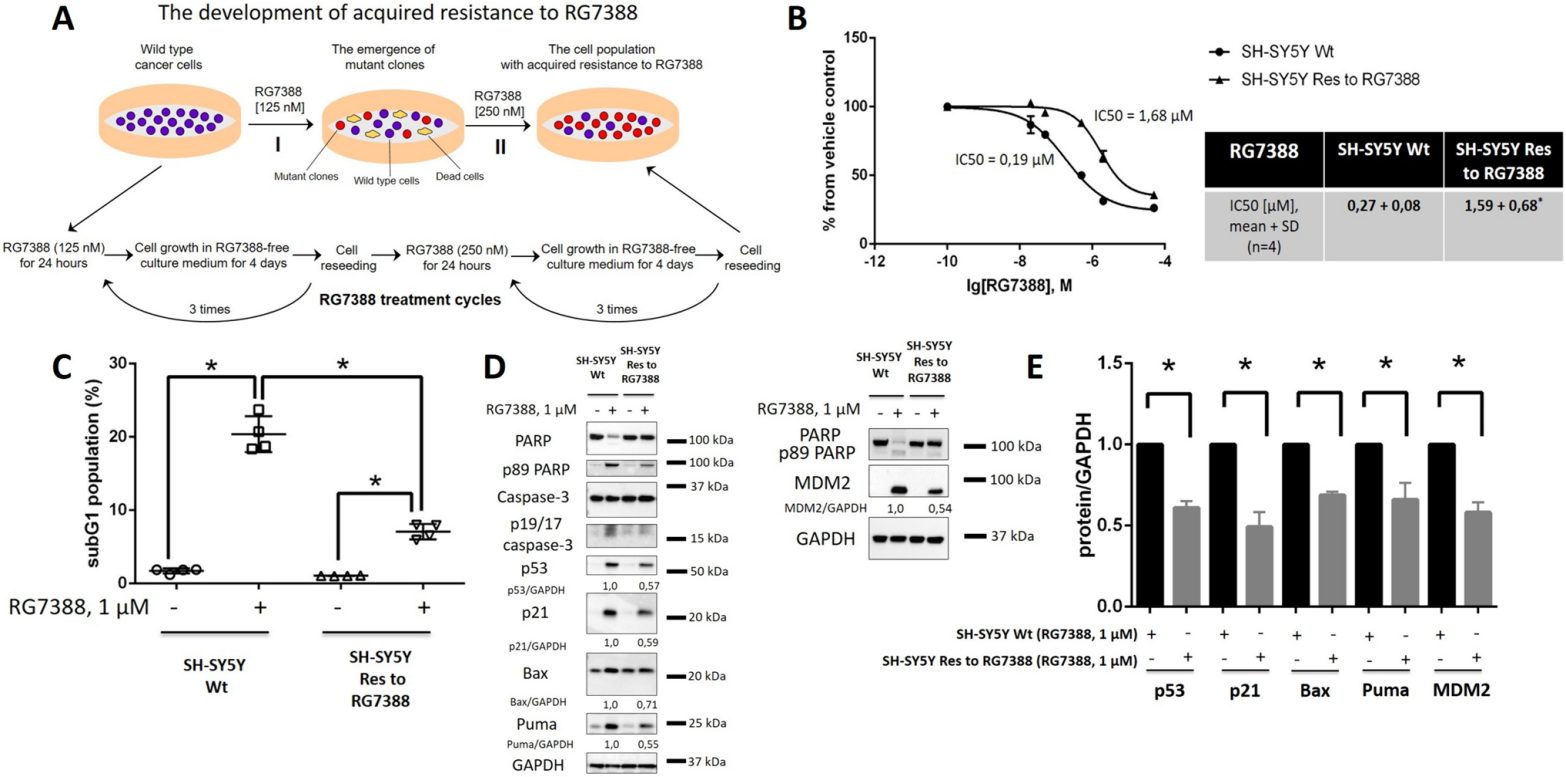
Generation of SH-SY5Y neuroblastoma cells resistant to RG7388 (SH-SY5Y Res).** A** Scheme of the development of acquired resistance to RG7388 in SH-SY5Y cells. **B** MTS cell viability assay of SH-SY5Y (parental and resistant cells) upon treatment with RG7388. SubG1 test (**C**) and WB analysis (**D**) of SH-SY5Y Wt and SH-SY5Y Res upon treatment with RG7388 at 1 µM. **E** Densitometric analysis of p53, p21, Bax, Puma, and MDM2 normalized to GAPDH. Results are presented as mean ± standard deviation (SD), *n* = 4 (Mann-Whitney U-test), *p < 0.05, *ns* not significant. GAPDH was used as a loading control. Incubation time: 24 h

To search for potential causes of acquired resistance to MDM2 inhibition in cancer cells, we developed SH-SY5Y neuroblastoma cells, which are characterized by lower sensitivity to RG7388 (SH-SY5Y Res) compared to parental wild-type cells (SH-SY5Y Wt). These cells were generated by cultivation of SH-SY5Y Wt cells with gradually increasing doses of RG7388, starting with 125 nM, which is a sub-IC50 dose of RG7388, and after three treatment cycles the dose of RG7388 was increased twice to the ~ IC50 dose (250 nM) (Fig. 1A, B). The resistance of the resulting cells was analyzed by MTS assay. The IC50 values were significantly augmented (~ 5–6 fold) in SH-SY5Y Res cells compared to SH-SY5Y Wt (Fig. 1B). The efficacy of apoptosis induction was estimated using the subG1 test, which reflects cell death intensity due to the accumulation of cells with the endonuclease-formed fragmented DNA [42]. The number of subG1 fractions was 2.5–3 times lower in resistant cells than in Wt cells (Fig. 1C). These results were supported by western blot (WB) analysis. For this purpose, the processing of caspase-3 and cleavage of its substrate PARP which are well-known apoptotic markers, were evaluated. The lower levels of full-length caspase-3 and PARP and higher levels of catalytically active fragments p19/17 of caspase-3 and p89 PARP correlated with apoptosis activation [43, 44]. The processing of both markers was essentially lower in SH-SY5Y Res in comparison to SH-SY5Y Wt cells (Fig. 1D). Similar results were obtained for parental and resistant cells over a wide range (0.25–4 µM) of RG7388 concentrations (Fig. S2). Altogether, these results confirm the presence of acquired resistance to MDM2 inhibition in the SH-SY5Y cell line.

**Fig. 2:**
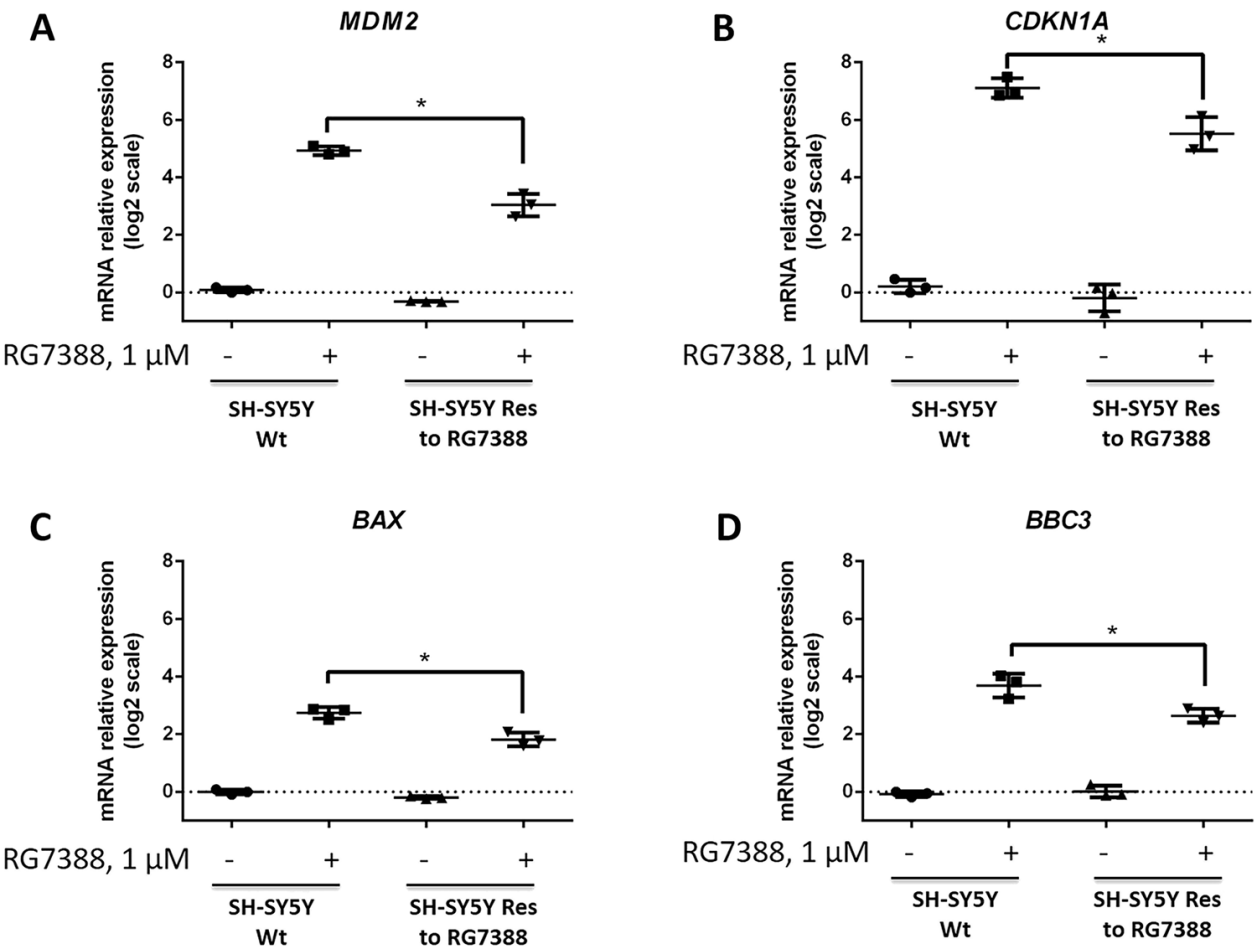
Analysis of p53 transcriptional activity in wild-type and RG7388-resistant neuroblastoma cells (SH-SY5Y Wt and SH-SY5Y Res, respectively). mRNA level of MDM2 (**A**—MDM2), p21 (**B**—CDKN1A), Bax (**C—**BAX), and Puma (**D—**BBC3) in Wt and resistant SH-SY5Y cells to RG7388 (1 µM, 24 h) treatment. TATA-binding protein (TBP) mRNA was used to normalize mRNA levels. Data are presented as individual datapoints from three independent repeats and mean ± standard deviation (SD) at log2 scale, two-tailed Student’s t-test with Welch’s correction, *p < 0.05

Importantly, upon RG7388 treatment, augmented levels of p53, MDM2, and p53-induced proteins (e.g., p21, Bax, and Puma), which are known biomarkers of the activity of MDM2 inhibitors, were observed [10]. Interestingly, the accumulation of these proteins in response to RG7388 treatment was essentially reduced in resistant cells in comparison with parental cells (Fig. 1D, E). Moreover, the levels of mRNA of MDM2 (*MDM2*), p21 (*CDKN1A*), Bax (*BAX*), and Puma (*BBC3*) were significantly decreased under the same conditions in SH-SY5Y Res cells in contrast with Wt cells (Fig. 2). Since p53 acts as a pivotal regulator of transcription, these results indicate impaired transcriptional activity of p53 in resistant SH-SY5Y cells.

### His193Arg p53 mutation affected its functions and led to acquired resistance to MDM2 inhibition

It is known that increased P-glycoprotein (P-gp) activity could be associated with the development of multiple drug resistance in tumor cells [45]. However, nutlins, MDM2 antagonists, were reported to inhibit P-gp and interrupt P-gp-mediated drug efflux [46]. Therefore, a P-gp inhibitor, Zosuquidar, was used in our study in combination with RG7388 to confirm this statement. According to the subG1 test and WB analysis, Zosuquidar could not enhance RG7388-induced apoptosis in resistant SH-SY5Y cells (Fig. S3), suggesting that the acquired resistance to RG7388 was not associated with changes in P-gp activity.

**Fig. 3:**
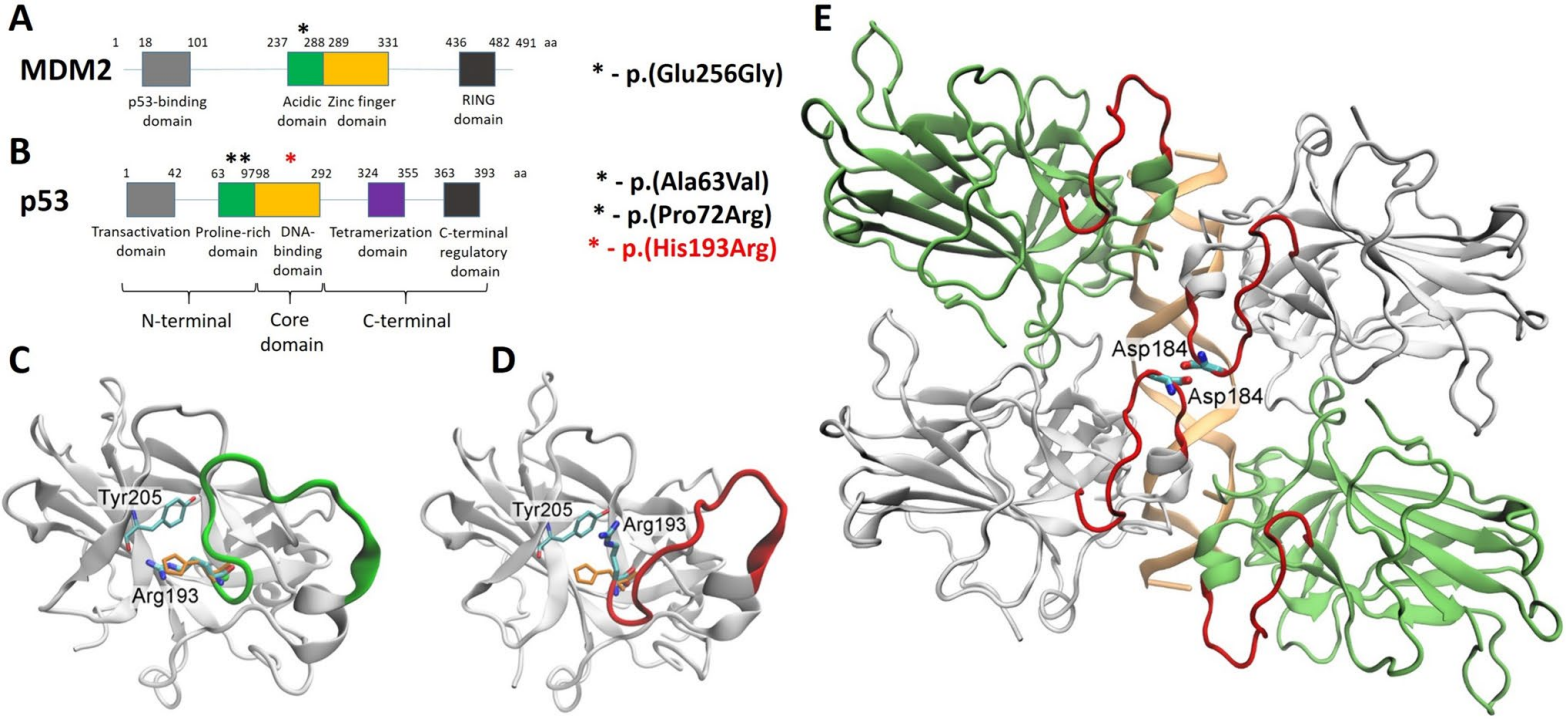
Analysis of the potential origins of acquired resistance to MDM2 inhibition. The structures of MDM2 (**A**) and p53 (**B**) proteins accordant with [51] and [52], respectively; *location of observed mutations. The major conformational change observed in the GaMD simulation of the His193Arg p53. **C** Initial structure of the mutant protein. The loop 182–194 is shown in green. **D** Protein structure after a conformational change. The loop 182–194 is shown in red. The coordinates of His193 in the Wt protein are shown in orange. Hydrogen atoms are omitted for clarity. **E** Unfavorable contacts between mutant p53 proteins in a modeled tetrameric assembly. A GaMD structure of the His193Arg mutant, shown in white or lime, was superimposed onto p53 subunits of the Wt p53-DNA complex (PDB ID 5mg7; DNA from the 5mg7 structure is shown in orange). The loop residues 182–194 are shown in red. Two neighboring p53 subunits form an unfavorable interaction between negatively charged Asp184 side chains

A decline in the accumulation of p53 and low expression of p53-induced proteins may result from the appearance of mutations in MDM2 protein, in particular, in a p53-binding pocket of this protein, which, in turn, interferes with the binding of MDM2 antagonists to their target. To test for this possibility, NGS sequencing of *MDM2* was performed. One mutation (Glu256Gly) in SH-SY5Y Res cells was detected in the acidic domain of MDM2 protein (Fig. 3A). However, according to molecular modeling, this mutation did not influence MDM2 stability and its interaction with p53 (data not shown).

It is possible that mutations in p53 could underlie its low stability and disturbed transcriptional activity. Based on NGS sequencing of *TP53*, three point mutations, which led to amino acid substitutions, were observed in the structure of the p53 protein (Fig. 3B). Two mutations (Ala63Val and Pro72Arg) were located in the proline-rich domain and did not influence p53 stability and function. Previously, Ala63Val was determined as a «neutral mutation» with no significant effect on p53 functional activity [47]. Notably, unlike other mutations, Pro72Arg was also detected in SH-SY5Y Wt cells. Moreover, Pro72Arg is a well-known and frequent Single Nucleotide Polymorphism (SNP) of *TP53*. Numerous studies have been devoted to the relation between this SNP and a high risk of tumor development, but this issue remains controversial [48–50].

At the same time, His193Arg, located in the DNA-binding domain, was able to affect p53 function in compliance with molecular modeling. It should be mentioned that mutations leading to the inactivation of the p53 protein can be divided into three classes: (i) disrupting direct contacts of protein with DNA, (ii) disturbing the stability of protein structure, and (iii) affecting the cooperative nature of p53-DNA binding [53]. As His193 does not interact directly with DNA, the His193Arg mutation may affect p53 stability and/or binding cooperativity. Models of Wt and mutant forms of p53 were constructed based on a crystal structure of the human p53 core domain. The His193 residue in the Wt protein was modeled in the uncharged form HIE, accounting for its hydrophobic environment formed by the Pro190 and Tyr205 residues (the calculated p*K*_a_ of His193 was found to be 5.7). cMD simulations of p53 conducted for 500 ns did not reveal any significant differences between the Wt and His193Arg structures. To accelerate the conformational sampling, a GaMD approach was applied (an advanced MD method for enhanced sampling of biomolecules that works by adding a harmonic boost potential to smooth the potential energy surface and reduce energy barriers) [54, 55]. In one of 500 ns GaMD production simulations of the His193Arg mutant, a major conformational change was observed: the Arg193 side chain moved away from its initial environment (formed by Pro190, Tyr205, and His214) at *t* ≈ 300 ns. At the same time, the His193 residue in the Wt protein (corresponding to Arg193 in the mutant) did not undergo significant conformational changes during GaMD simulations.

The Arg193 mobility in the p53 mutant was accompanied by the formation of a more favorable cation-*π* interaction between the Arg193 and Tyr205 side chains and with the transition of a nearby loop, comprising residues 182–194, to a new conformation (Fig. 3C, D; Fig. S1B). The loop 182–194 is a part of the so-called loop L2 in the p53 structure [56], and its overall conformation apparently depends on the position of the bulky side chain of Arg193. The concerted displacement of Arg193 and other loop residues (Asp184, Ser185, Leu188, Ala189, Pro190) produced a p53 interface that was different from the wild-type one observed in crystal structures (e.g., PDB ID 5mg7).

Next, we demonstrated a modeled tetrameric assembly of mutant p53 proteins in which p53 conformations obtained from the GaMD simulation were superimposed onto a crystal structure of the Wt p53-DNA complex (Fig. 3E). Due to the mutant loop conformation, two neighboring p53 proteins may form unfavorable intermolecular contacts (in particular, between two negatively charged aspartate residues), thus hindering tetramerization. Notably, intact p53 binds DNA as a tetramer in a highly cooperative manner [57–59]. Positive cooperativity relies on the stabilization of the p53 assembly by interactions between monomers, and Glu180 and Arg181 are the key residues that mediate the reciprocal electrostatic interaction between the p53 core domains [53, 60]. Molecular modeling demonstrated that the His193Arg mutation is likely to be a “cooperative” mutation that destabilizes the tetrameric p53-DNA complex due to an unusual conformational state of the loop 182–194, thus affecting the transcriptional activity of p53 (Fig. 3E).

These findings are in agreement with the existing data showing that His193Arg p53 protein possesses diminished DNA binding activity [61]. Interestingly, His193Arg p53 was able to retain its transactivation ability mainly at sub-physiological temperatures (between + 24 and + 30 °C) and display weak activation at + 37 °C [62]. Furthermore, this mutation was reported to hinder the stability of the p53 protein due to the loss of a salt bridge that resulted in its low protein concentration [63]. Taken together, the evidence shows that His193Arg caused a decline in the stability of p53 and its tetrameric complex with DNA, which explains the decreased accumulation of p53 and p53-induced proteins in response to RG7388 treatment in resistant cells compared to Wt cells presented above. Hence, deleterious p53 mutations lead to the development of non-response to MDM2 antagonists, which is in agreement with other reports [64–67].

Next, to examine the frequency and distribution of His193Arg in various types of cancer, we used two open databases of *TP53* mutations. According to the TP53 Database ((R20, July 2019): https://tp53.cancer.gov, [68]), this mutation (*n* = 101) was found most frequently in ovarian (*n* = 18), breast (*n* = 13), lung (*n* = 12), and esophageal tumors (*n* = 11) (Table 1).

**Table 1:** Frequency of occurrence of the p53 mutation (His193Arg) in various tumor types according to the TP53 database (R20, July 2019): https://tp53.cancer.gov

Tumor distribution	*N* = 101
Ovary	18
Breast	13
Lung	12
Esophagus	11
Hematopoietic system	6
Head and neck	5
Stomach	5
Brain	4
Bladder	4
Lymph nodes	3
Nasal cavity	2
Skin	2
Rectum	2
Liver	2
Corpus uteri	1
Colon	1
Colorectum	1
Uterus	1
Hypopharynx	1
Oropharynx	1
Prostate	1
Mouth (floor)	1
Pancreas	1
Larynx	1
Renal pelvis	1
Cervix uteri	1

At the same time, His193Arg p53 was present in 0.22% (*n* = 157) of cases (Table 2), and the greatest prevalence was observed in patients with breast invasive ductal carcinoma (*n* = 23), high-grade ovarian serous adenocarcinoma (*n* = 22), lung adenocarcinoma (*n* = 19), and pancreatic adenocarcinoma (*n* = 13), corresponding to AACR GENIE (https://www.mycancergenome.org, [69]). Altogether, His193Arg p53 is not unique to the selected neuroblastoma model. By contrast, this mutation, detected in many tumors, is considered to be clinically significant.

**Table 2:** Prevalence of the p53 mutation (His193Arg) in cancer patients according to the AACR GENIE (https://www.mycancergenome.org)

Disease	*N* = 157
Breast invasive ductal carcinoma	23
High-grade ovarian serous adenocarcinoma	22
Lung adenocarcinoma	19
Pancreatic adenocarcinoma	13
Glioblastoma	7
Conventional glioblastoma multiforme	5
Endometrial serous adenocarcinoma	5
Anaplastic astrocytoma	5
Colon adenocarcinoma	5
Colorectal adenocarcinoma	4
Esophageal adenocarcinoma	4
Astrocytoma	4
Invasive breast carcinoma	4
Prostate adenocarcinoma	3
Myelodysplastic syndromes	3
Endometrial endometrioid adenocarcinoma	3
Sarcoma, NOS (not otherwise specified)	2
Ovarian serous adenocarcinoma	2
Small cell lung carcinoma	2
Ovarian carcinosarcoma	2
Oral cavity squamous cell carcinoma	2
High grade fallopian tube serous adenocarcinoma	2
Leiomyosarcoma	2
Laryngeal squamous cell carcinoma	2
Gliosarcoma	2
Diffuse glioma	2
Diffuse astrocytoma	2
Cancer of unknown primary	2
Bladder urothelial carcinoma	2
Unknown	2

### SH-SY5Y Res cells possessed lower sensitivity to DNA-damaging agents in vitro and in vivo compared to SH-SY5Y Wt cells

**Fig. 4:**
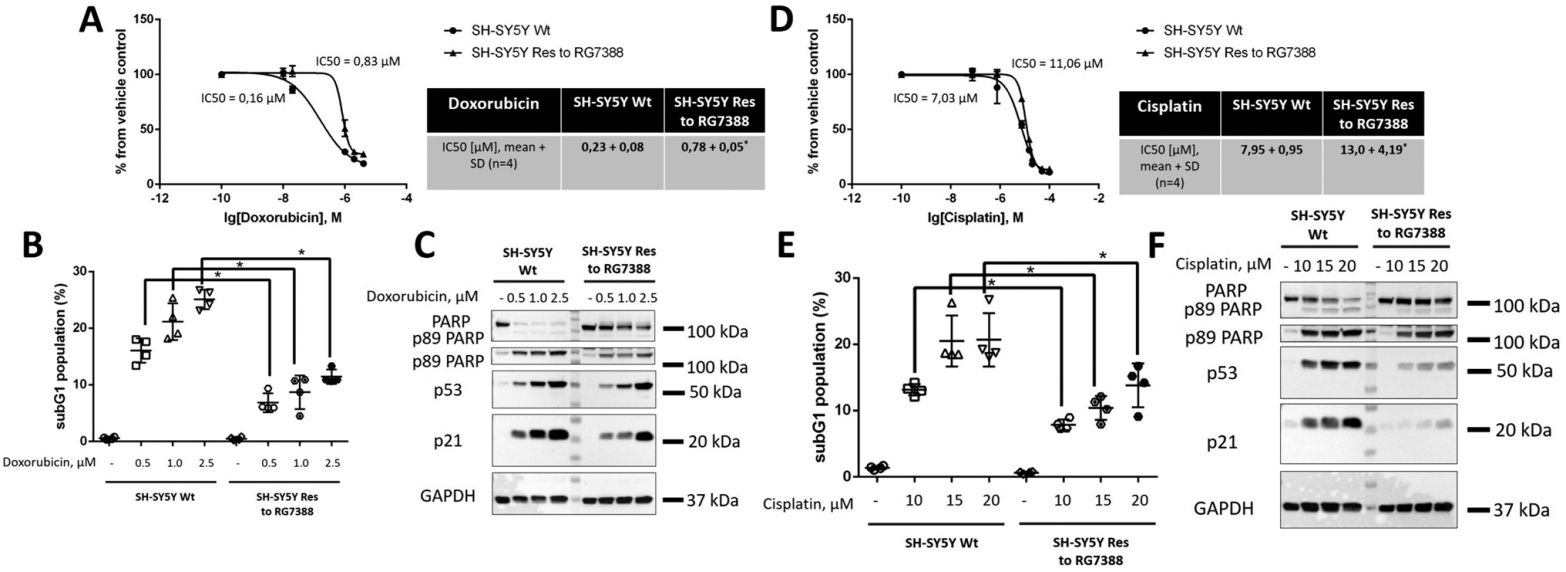
Analysis of the efficiency of Doxorubicin and Cisplatin alone in wild-type and RG7388-resistant neuroblastoma cells (SH-SY5Y Wt and SH-SY5Y Res, respectively). MTS cell viability assay (**A**,** D**), subG1 test (**B**,** E**), and WB (**C**,** F**) analysis of SH-SY5Y (parental and resistant cells) upon treatment with Doxorubicin (**A-C**) and Cisplatin (**D-F**). Results are presented as mean ± standard deviation (SD), *n* = 4 (Mann-Whitney U-test), *p < 0.05, *ns* not significant. GAPDH was used as a loading control. Incubation time: 24 h

Resistance to MDM2 inhibition may be accompanied by the development of insensitivity to chemotherapeutics [65], especially DNA-damaging agents, since the p53 protein mediates apoptosis induced by genotoxic stress [70]. Firstly, we evaluated the efficacy of Doxorubicin and Cisplatin alone, which are commonly used in neuroblastoma therapy in vitro models of resistance to MDM2 inhibition [71] (Fig. 4). According to the MTS assay, the IC50 values of Doxorubicin and Cisplatin increased prominently (~ 3.5 and 1.5 fold, respectively) in resistant cells compared to SH-SY5Y Wt cells (Fig. 4A, D). Moreover, both drugs abated apoptosis in resistant cells relative to parental cells as evidenced by the decline in the subG1 population (Fig. 4B, E) and PARP cleavage (Fig. 4C, F). Thus, Doxorubicin and Cisplatin as single agents have shown low efficacy in vitro in SH-SY5Y Res cells based on three independent experimental approaches.

**Fig. 5:**
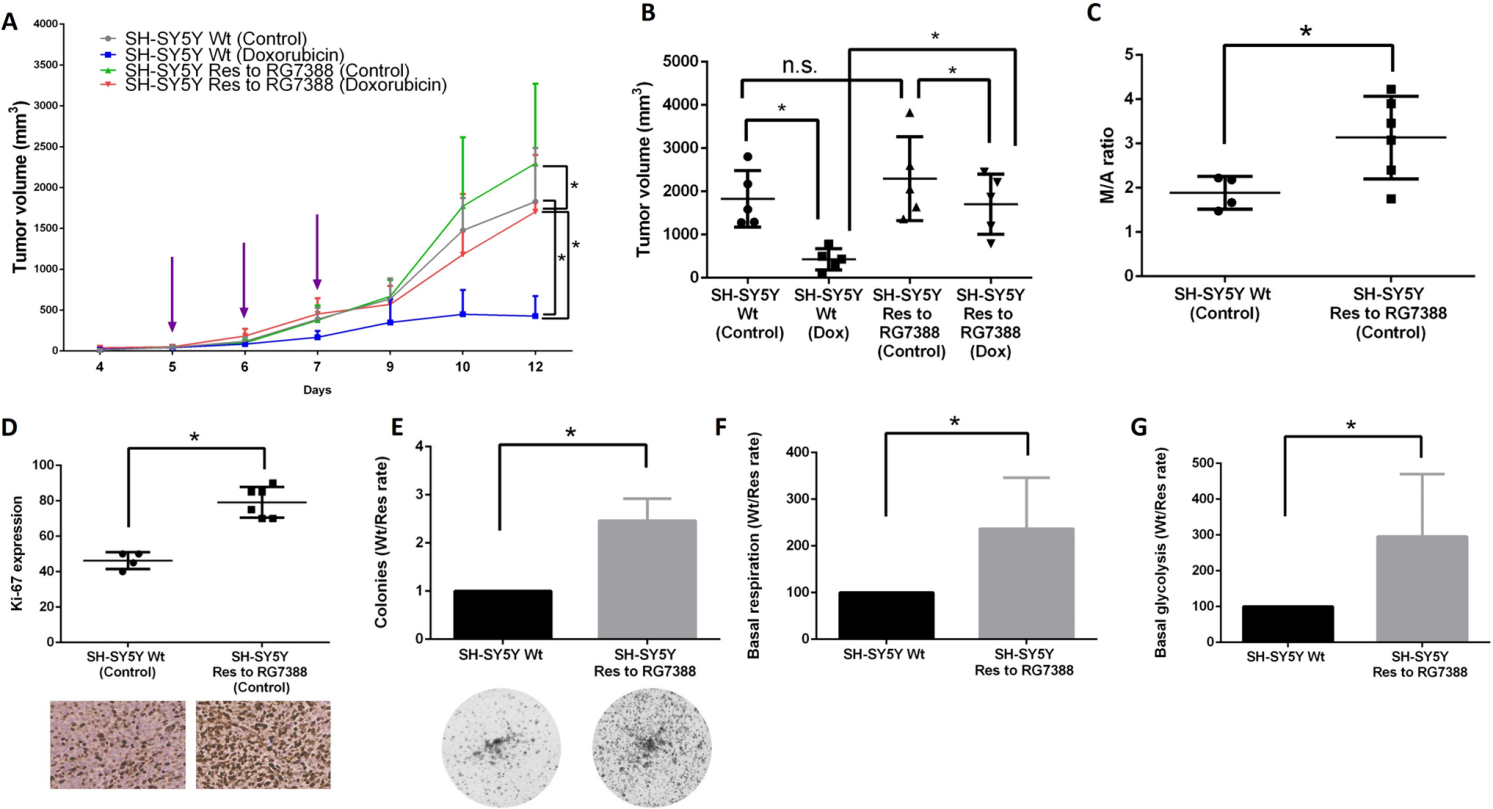
Analysis of the efficiency of Doxorubicin alone in a mouse xenograft model of acquired resistance to RG7388 (**A**, **B**) and evaluation of the proliferative and metabolic activities of RG7388-resistant (SH-SY5Y Res) compared to wild-type (SH-SY5Y Wt) neuroblastoma cells in vivo **(C**,** D) **and in vitro **(E**–**G)**. Tumor growth dynamics (**A**) and tumor volume at day 12 (**B**) of the «Control» and «Doxorubicin» groups (*n* = 5 for each group). Violet arrows indicate doxorubicin injections (3 mg/kg/day). M/A ratio (**C**) and Ki-67 expression (**D**) in tumors bearing Wt and resistant SH-SY5Y cells obtained from mouse xenografts. **E** Clonogenic assay of parental and resistant SH-SY5Y cells. The data are presented as a rate (Wt/Res cells) of the number of colonies, normalized to Wt cells (*n* = 4). **F**–**G** Assessment of the basal respiration (**F**) and glycolysis (**G**) rates. The data are presented as a rate (Wt/Res cells), normalized to Wt cells (*n* = 4). Results are presented as mean ± standard deviation (SD); Mann-Whitney U-test, *p < 0.05, *ns* not significant; *Dox *Doxorubicin. Details are displayed in the sections «Materials and Methods», «Results» and Supplementary Information (SI)

Next, Doxorubicin was tested alone in an in vivo mouse xenograft model of acquired resistance to RG7388. According to tumor growth dynamics (Fig. 5A) and tumor volume at day 12 (the termination of the experiment) (Fig. 5B), in four experimental groups (SH-SY5Y Wt «Control», SH-SY5Y Wt «Doxorubicin», SH-SY5Y Res to RG7388 «Control», and SH-SY5Y Res to RG7388 «Doxorubicin»; *n* = 5 for each group), Doxorubicin was able to suppress tumor growth both in mice bearing parental cells and in those bearing resistant cells, but the anticancer activity of Doxorubicin was significantly weaker in the in vivo model of resistance to MDM2 inhibition compared to the animals bearing tumors induced by Wt neuroblastoma cells. No statistical differences were observed between the two «Control» groups.

An additional set of animals (two extra «Control» groups: SH-SY5Y Wt (*n* = 4) and SH-SY5Y Res (*n* = 6) cells) was used for evaluation of the proliferative activity of tumors via analysis of the M/A ratio (Fig. 5C) and Ki-67 expression (Fig. 5D). Tumors from the SH-SY5Y Res «Control» group were found to have higher M/A ratios (~ 1.5 fold) and possess more Ki-67-positive cells (~ 2.0 fold) as compared with tumors from SH-SY5Y Wt group (Fig. 5C, D; Figs. S4, S5; Table S4). Furthermore, these results correlated with our in vitro data: the resistant cells were more clonogenic than Wt cells (Fig. 5E). The differences in proliferative activity between SH-SY5Y Wt and SH-SY5Y Res cells may be explained by changes in the metabolism of the latter. Based on the basal respiration and glycolysis tests, resistant cells were much more metabolically active than parental cells (Fig. 5F, G). Thereby, non-response to MDM2 inhibition promotes the generation of more aggressive and faster-proliferating cancer cells.

### Cisplatin or BH3-mimetics in combination with RG7388 were able to partially overcome acquired resistance to MDM2 inhibition in neuroblastoma cells

**Fig. 6:**
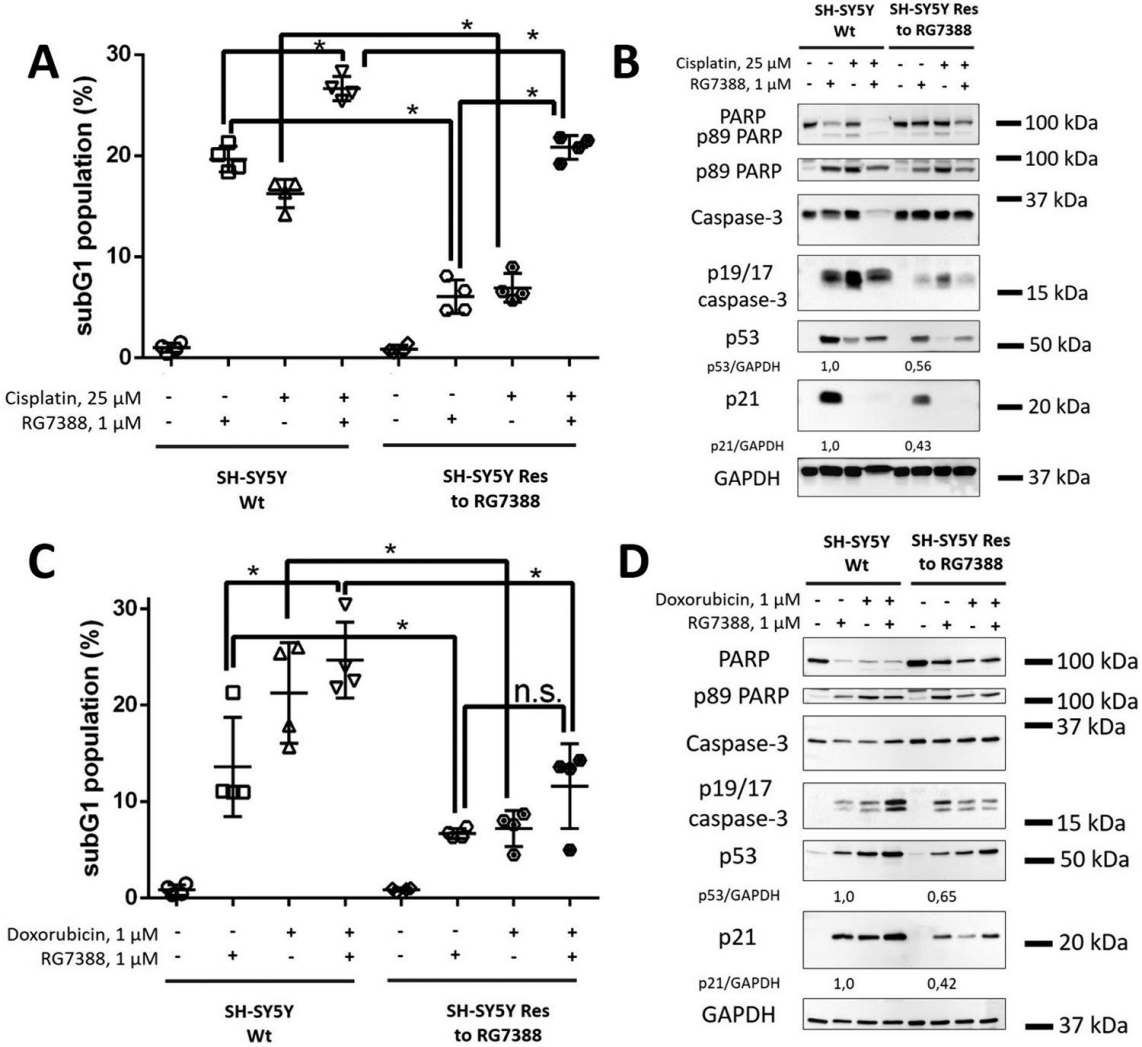
Analysis of the efficiency of Cisplatin and Doxorubicin in combination with RG7388 in SH-SY5Y wild-type and resistant cells. SubG1 test (**A**,** C**) and WB analysis (**B**,** D**) of SH-SY5Y (parental and resistant cells, respectively) in response to Cisplatin (25 µM) (**A**,** B**) and Doxorubicin (1 µM) (**C**,** D**). Results are presented as mean ± standard deviation (SD), *n* = 4 (Mann-Whitney U-test), *p < 0.05, *ns* not significant. GAPDH was used as a loading control. Incubation time: 24 h

A promising therapeutic strategy is the use of drug combinations to prevent and overcome acquired resistance to monotherapy. The drugs of various groups were shown to be effective in combination with MDM2 antagonists [21]. Therefore, firstly, we decided to assess whether DNA-damaging agents (Doxorubicin and Cisplatin) were able to overcome acquired resistance to MDM2 inhibition coupled with RG7388 (Fig. 6). According to the subG1 and WB data, Doxorubicin (1 µM) and Cisplatin (25 µM) enhanced RG7388-mediated apoptosis in SH-SY5Y Wt cells. However, only Cisplatin (25 µM) + RG7388 (1 µM) could partially overcome non-response to MDM2 inhibition in SH-SY5Y Res cells (Fig. 6A, B), while Doxorubicin (1 µM) + RG7388 (1 µM) slightly increased cell death induced by RG7388 (Fig. 6C, D).

Earlier, we demonstrated that both drugs possessed low efficacy alone in SH-SY5Y Res cells. At the same time, Cisplatin, unlike Doxorubicin, combined with RG7388, partially overcame resistance to this inhibitor in neuroblastoma cells.

**Fig. 7:**
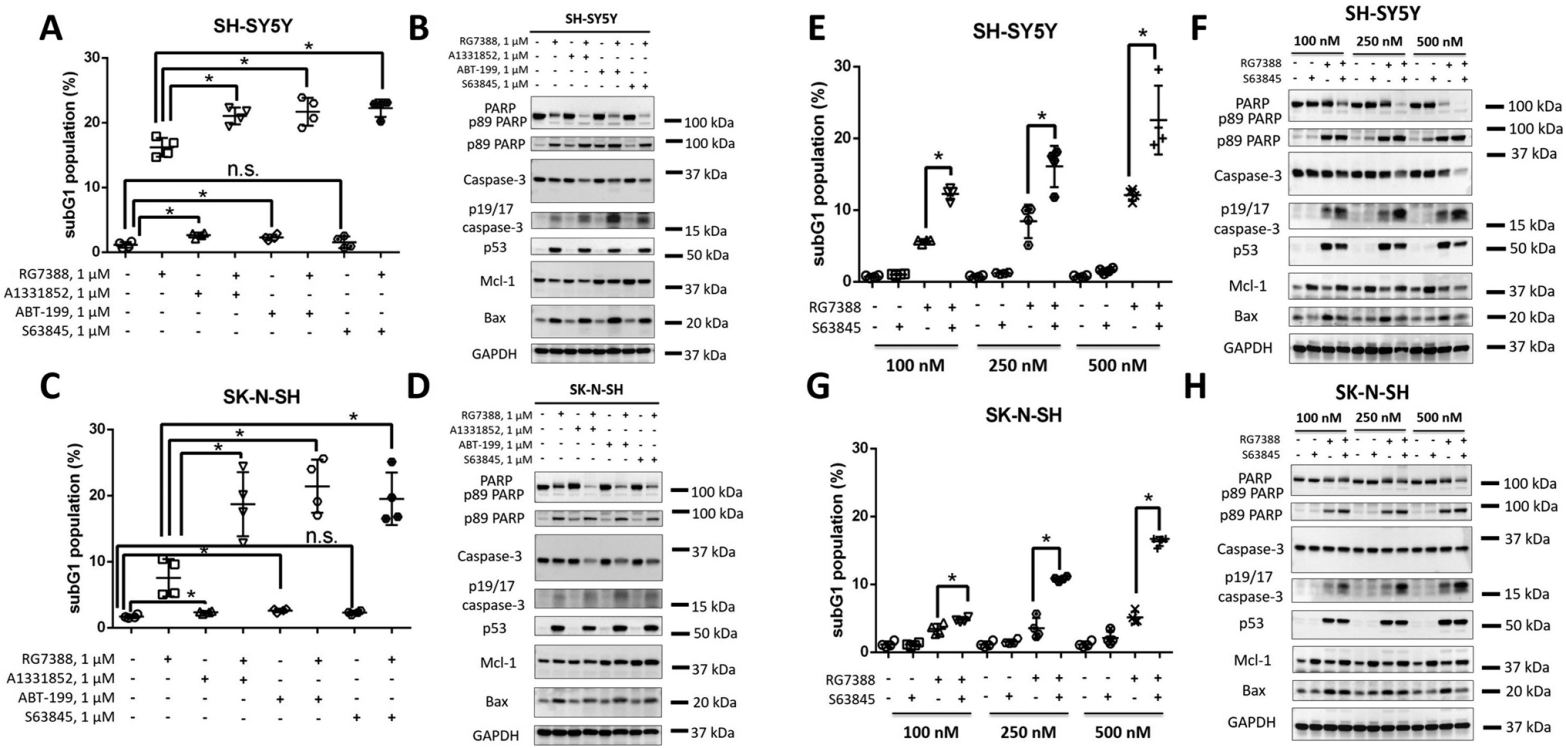
Analysis of the efficiency of concomitant inhibition of Bcl-xL (A1331852)/Bcl-2 (ABT-199)/Mcl-2 (S63845) and MDM2 (RG7388) in SH-SY5Y and SK-N-SH cells. **A**,** C** SubG1 test and **B**,** D** WB analysis of SH-SY5Y (**A**,** B**) and SK-N-SH (**C**,** D**) cells upon treatment with various BH3-mimetics and RG7388. All compounds were used at a concentration of 1 µM. **E**,** G** subG1 test and **F**,** H** WB analysis of SH-SY5Y (**E**,** F**) and SK-N-SH (**G**,** H**) cells in response to co-targeting of Mcl-1 and MDM2. Cells were treated with either or both of 100–500 nM S63845 and 100–500 nM RG7388. Results are presented as mean ± standard deviation (SD), *n* = 4 (Mann-Whitney U-test), *p < 0.05, *ns* not significant. GAPDH was used as a loading control. Incubation time: 24 h

It has been shown that several other targeted drugs could have synergistic effects along with MDM2 antagonists [12, 21]. As mentioned above, the BH3 mimetic ABT-199/Venetoclax (a Bcl-2 inhibitor), together with RG7388, demonstrated promising results in vitro and in vivo [25], and now this combination is being evaluated in clinical trials in patients with solid and hematological malignancies (NCT04029688). Keeping that in mind, we decided to investigate whether this effect is only specific to the co-targeting of Bcl-2 and MDM2 or whether the other BH3 mimetics could also synergize with RG7388. In this regard, we performed an analysis of the efficiency of concomitant inhibition of Bcl-2 (ABT-199)/Bcl-xL, (A1331852)/Mcl-2 (S63845), and MDM2 (RG7388) in SH-SY5Y and another neuroblastoma cell line-SK-N-SH (Fig. 7). The latter also contains p53 Wt, but SK-N-SH is less sensitive to MDM2 inhibition compared to SH-SY5Y [14, 72]. All BH3-mimetics possessed low cytotoxicity as single agents: the Mcl-1 inhibitor did not induce cell death, and Bcl-2 or Bcl-xL inhibitors slightly increased apoptosis in SH-SY5Y and SK-N-SH cells. However, all compounds were able to significantly enhance RG7388-mediated apoptosis according to the subG1 test (Fig. 7A, C). These results were supported by WB data (Fig. 7B, D). Thus, all selected BH3-mimetics were found to synergize with the MDM2 antagonist RG7388.

As mentioned above, nowadays, the Bcl-2 inhibitor ABT-199/Venetoclax has been approved by the FDA for use in humans, and several Mcl-1 inhibitors are being investigated in clinical trials. However, the use of Bcl-xL inhibitors is hindered by the development of severe side effects (e.g., thrombocytopenia) [22, 23]. Hence, the co-inhibition of Mcl-1/MDM2 along with Bcl-2/MDM2 may have therapeutic potential in cancer therapy. To confirm this suggestion, lower doses of S63845 and RG7388 were used in further experiments with SH-SY5Y and SK-N-SH cells. Firstly, the rate of RG7388-mediated apoptosis at 1 µM was comparable to apoptosis levels induced by 500 nM RG7388 + 500 nM S63845 (Fig. S6A–D). Secondly, S63845 starting from 100 nM strongly enhanced the rate of RG7388-mediated apoptosis at 1 µM (Fig. S6E–H). Thirdly, RG7388 + S63845 at nanomolar concentrations from 100 nM led to a synergistic effect in SH-SY5Y and SK-N-SH cells according to the subG1 test and WB results (Fig. 7E–H). Taken together, these findings give a rationale for further evaluation of concomitant inhibition of Mcl-1 and MDM2.

**Fig. 8:**
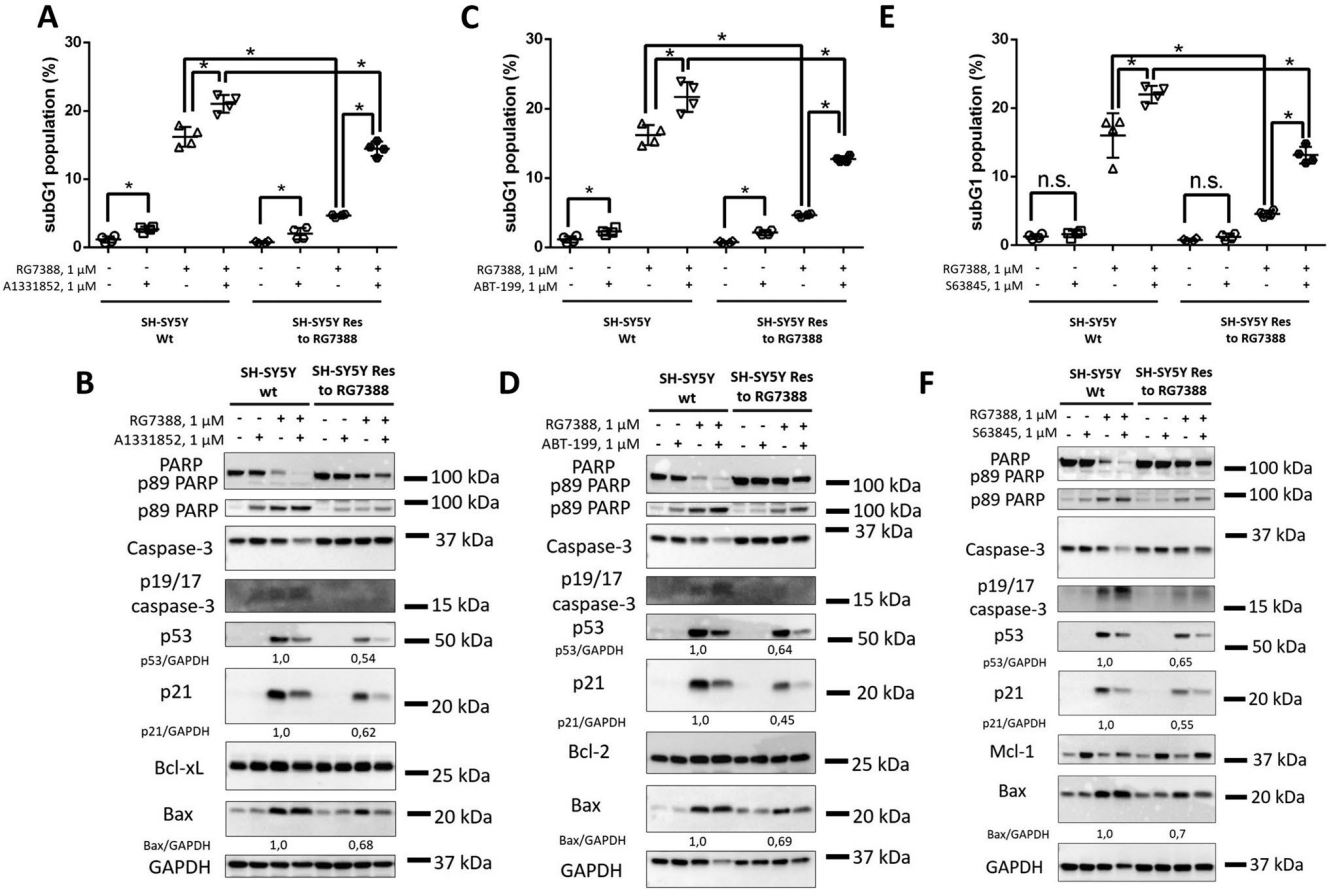
Analysis of the efficiency of concomitant inhibition of Bcl-xL (A1331852)/Bcl-2 (ABT-199)/Mcl-2 (S63845) and MDM2 (RG7388) in RG7388-resistant neuroblastoma cells (SH-SY5Y Res). SubG1 test (**A**,** C**,** E**) and WB (**B**,** D**,** F**) analysis of SH-SY5Y (parental and resistant cells) in response to A1331852 + RG7388 (**A**,** B**), ABT-199 + RG7388 (**C**,** D**) and S63845 + RG7388 (**E**,** F**). All compounds were used at 1 µM. Results are presented as mean ± standard deviation (SD), *n* = 4 (Mann-Whitney U-test), *p < 0.05, *ns* not significant. GAPDH was used as a loading control. Incubation time: 24 h

Finally, we tested whether selected BH3-mimetics combined with RG7388 can overcome resistance to MDM2 inhibition (Fig. 8). According to the subG1 and WB analysis, all drugs significantly increased RG7388-mediated cell death in resistant cells, but the apoptosis rate upon administration of the BH3 mimetic with the MDM2 antagonist was remarkably lower in SH-SY5Y Res cells than in parental cells in all cases. Hence, these compounds only partially overcame resistance to MDM2 inhibition.

## Discussion

**Fig. 9:**
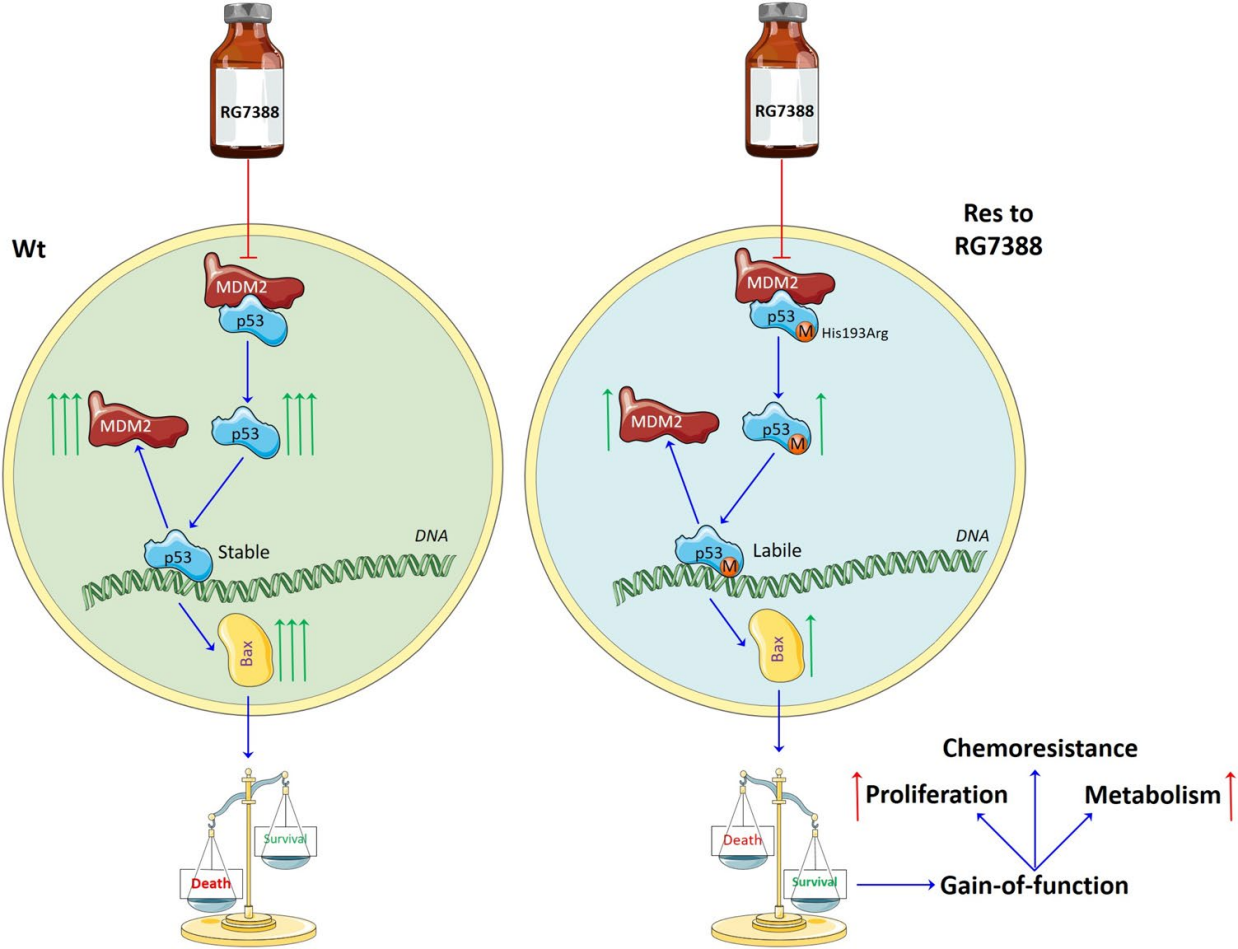
Differences between wild-type and resistant cells upon RG7388 treatment. M—mutated p53 protein

**Fig. 10:**
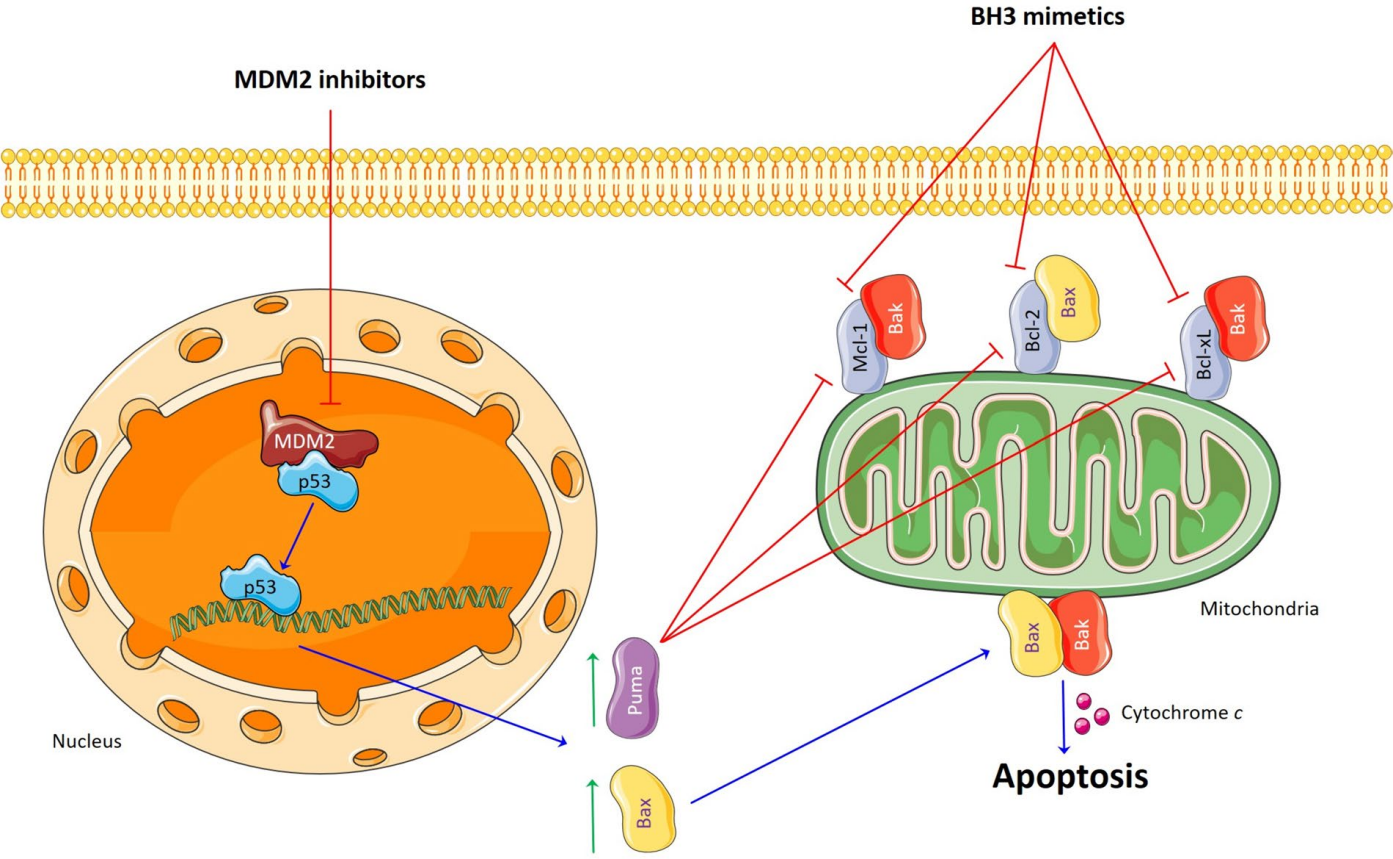
Facilitation of apoptosis induced by MDM2 inhibitors and BH3-mimetics in cancer cells

Drug resistance is one of the key challenges in medicine, especially in cancer therapy. This problem is also inherent in MDM2 inhibitors, which are currently under extensive evaluation [16]. Notably, MDM2 gene amplification was found in various tumor types, including neuroblastoma [73, 74]. Moreover, MDM2 overexpression in malignant cells is associated with an unfavorable prognosis for cancer patients [75]. The main differences between SH-SY5Y Wt and SH-SY5Y Res cells are summarized in Fig. 9. Acquired resistance to RG7388 was associated with the appearance of a p53 mutation (His193Arg) in the neuroblastoma model analyzed in this study. This mutation, characterized in detail, was detected in numerous human malignancies, led to decreased accumulation of p53 upon RG7388 treatment, and diminished the stability of the p53 tetrameric complex with DNA. As a result, it impaired the transcriptional activity of p53 and decreased the levels of p53-induced proapoptotic proteins, which finally caused a switching of cell fate from apoptosis to uncontrolled proliferation and survival in resistant cells compared to SH-SY5Y Wt cells. Moreover, the «gain-of-function» phenomenon was observed in SH-SY5Y Res cells. In this case, cells bearing mutant p53 protein not only abolish its onco-suppressive functions but also acquire additional oncogenic potential that promotes tumor development and progression [76, 77]. We found that resistant cells were more proliferative and metabolically active than Wt cells. Furthermore, they decreased their susceptibility to chemotherapeutics alone in both in vitro and in vivo models (Fig. 10). Hence, the acquisition of p53 mutations could lead to poor prognosis in cancer patients. At the same time, their presence may be an important biomarker in clinical research, which can predict the response to anticancer chemo- and target therapy.

Taken together, the evidence indicates that MDM2 is a prospective drug target. However, 20 years after the appearance of nutlin, no MDM2 inhibitor has been approved for the treatment of cancer patients. Several trials were discontinued due to the development of side effects and low drug efficacy [12, 13, 78, 79]. The latter is probably associated with the presence of deleterious p53 mutations that affect functional protein activity. Therefore, combined therapy using MDM2 inhibitors at low doses may lead to therapeutic benefits. Above all, this approach could prevent the development of resistance to MDM2 antagonists and increase their antitumor efficacy. We demonstrated that not only ABT-199 is able to enhance RG7388-mediated apoptosis, but another BH3-mimetic, namely S63845 (a Mcl-1 inhibitor), can synergize with RG7388. The mechanism underlying the enhancement of apoptosis by BH3-mimetics and MDM2 antagonists is presented in Fig. 10.

On the one hand, the addition of BH3-mimetics leads to the destruction of complexes between anti- and proapoptotic proteins of the Bcl-2 family and an increase in the levels of «unbound» effector proteins (Bak and Bax) [22, 23]. On the other hand, the action of RG7388 results in a block of MDM2 and accumulation of p53, which, in turn, elevates the levels of p53-induced effector (e.g., Bax) and regulatory (e.g., Puma) proapoptotic proteins [12]. It should be noted that regulatory members of the Bcl-2 family can neutralize prosurvival and activate proapoptotic proteins, and effectors which also participate in the permeabilization of the outer mitochondria membrane [22, 23]. Altogether, BH3-mimetics along with MDM2 antagonists promote the induction of the mitochondrial pathway of apoptosis in cancer cells. Furthermore, combined therapy could overcome acquired resistance to MDM2 inhibition. For instance, we found that DNA-damaging agents (e.g., Cisplatin) or BH3-mimetics partially restored sensitivity of resistant SH-SY5Y cells to RG7388.

It should be mentioned that resistance to MDM2 inhibition is not always associated with p53 mutations. Disturbances in regulatory pathways may be causes or consequences of non-response to MDM2 inhibition. Recently, it was reported that acquired resistance to RG7388 in glioblastoma cells was mediated by altered IGFBP1 expression and a disrupted ERK1/2 signaling cascade that was overcome by the MEK inhibitor Trametinib [66]. Moreover, various cytokines and chemokines involved in tumor invasion and epithelial-mesenchymal transition (EMT) were found to be increased in nutlin-resistant cells [80]. Additionally, ferroptosis (another type of PCD) inhibition was reported to contribute nutlins’ resistance in osteosarcoma cells [81]. Therefore, further investigations are required to comprehend the reasons of resistance to MDM2 antagonists and to discover novel drug combinations, which could promote their therapeutic benefit and approval in the future.

## Supplementary Information

Below is the link to the electronic supplementary material.

Supplementary material 1 (DOCX 1603 kb)

## Data Availability

No datasets were generated or analysed during the current study.

## References

[1] Levine AJ (1997) p53, the cellular gatekeeper for growth and division. Cell 88:323–331. 10.1016/s0092-8674(00)81871-1 9039259 10.1016/s0092-8674(00)81871-1

[2] Bates S, Vousden KH (1996) p53 in signaling checkpoint arrest or apoptosis. Curr Opin Genet Dev 6:12–18. 10.1016/s0959-437x(96)90004-0 8791489 10.1016/s0959-437x(96)90004-0

[3] Harris CC (1996) Structure and function of the p53 tumor suppressor gene: clues for rational cancer therapeutic strategies. J Natl Cancer Inst 88:1442–1455. 10.1093/jnci/88.20.1442 8841019 10.1093/jnci/88.20.1442

[4] Liu J, Zhang C, Hu W, Feng Z (2019) Tumor suppressor p53 and metabolism. J Mol Cell Biol 11:284–292. 10.1093/jmcb/mjy070 30500901 10.1093/jmcb/mjy070 PMC6487777

[5] Zhou X, Hao Q, Lu H (2019) Mutant p53 in cancer therapy-the barrier or the path. J Mol Cell Biol 11:293–305. 10.1093/jmcb/mjy072 30508182 10.1093/jmcb/mjy072 PMC6487791

[6] Nag S, Qin J, Srivenugopal KS et al (2013) The MDM2-p53 pathway revisited. J Biomed Res 27:254–271. 10.7555/JBR.27.20130030 23885265 10.7555/JBR.27.20130030 PMC3721034

[7] Shadfan M, Lopez-Pajares V, Yuan Z-M (2012) MDM2 and MDMX: alone and together in regulation of p53. Transl Cancer Res 1:88–89 23002429 PMC3448287

[8] Bazanov DR, Pervushin NV, Savin EV et al (2021) Sulfonamide derivatives of cis-imidazolines as potent p53-MDM2/MDMX protein-protein interaction inhibitors. Med Chem Res 30:2216–2227. 10.1007/s00044-021-02802-w

[9] Vousden KH, Lu X (2002) Live or let die: the cell’s response to p53. Nat Rev Cancer 2:594–604. 10.1038/nrc864 12154352 10.1038/nrc864

[10] Vassilev LT, Vu BT, Graves B et al (2004) In vivo activation of the p53 pathway by small-molecule antagonists of MDM2. Science 303:844–848. 10.1126/science.1092472 14704432 10.1126/science.1092472

[11] Bhatia N, Khator R, Kulkarni S et al (2023) Recent advancements in the discovery of MDM2/MDM2-p53 Interaction inhibitors for the treatment of cancer. Curr Med Chem 30:3668–3701. 10.2174/0929867330666221114103924 37190755 10.2174/0929867330666221114103924

[12] Fallatah MMJ, Law FV, Chow WA, Kaiser P (2023) Small-molecule correctors and stabilizers to target p53. Trends Pharmacol Sci 44:274–289. 10.1016/j.tips.2023.02.007 36964053 10.1016/j.tips.2023.02.007 PMC10511064

[13] Hassin O, Oren M (2023) Drugging p53 in cancer: one protein, many targets. Nat Rev Drug Discov 22:127–144. 10.1038/s41573-022-00571-8 36216888 10.1038/s41573-022-00571-8 PMC9549847

[14] Bazanov DR, Pervushin NV, Savin EV et al (2022) Synthetic design and biological evaluation of New p53-MDM2 interaction inhibitors based on imidazoline core. Pharmaceuticals (Basel) 15:444. 10.3390/ph15040444 35455441 10.3390/ph15040444 PMC9027661

[15] Bazanov DR, Pervushin NV, Savitskaya VY et al (2019) 2,4,5-Tris(alkoxyaryl)imidazoline derivatives as potent scaffold for novel p53-MDM2 interaction inhibitors: design, synthesis, and biological evaluation. Bioorg Med Chem Lett 29:2364–2368. 10.1016/j.bmcl.2019.06.007 31196710 10.1016/j.bmcl.2019.06.007

[16] Haronikova L, Bonczek O, Zatloukalova P et al (2021) Resistance mechanisms to inhibitors of p53-MDM2 interactions in cancer therapy: can we overcome them? Cell Mol Biol Lett 26:53. 10.1186/s11658-021-00293-6 34911439 10.1186/s11658-021-00293-6 PMC8903693

[17] Zafar A, Wang W, Liu G et al (2021) Targeting the p53-MDM2 pathway for neuroblastoma therapy: rays of hope. Cancer Lett 496:16–29. 10.1016/j.canlet.2020.09.023 33007410 10.1016/j.canlet.2020.09.023 PMC8351219

[18] Van Maerken T, Rihani A, Van Goethem A et al (2014) Pharmacologic activation of wild-type p53 by nutlin therapy in childhood cancer. Cancer Lett 344:157–165. 10.1016/j.canlet.2013.11.002 24262662 10.1016/j.canlet.2013.11.002

[19] Lakoma A, Barbieri E, Agarwal S et al (2015) The MDM2 small-molecule inhibitor RG7388 leads to potent tumor inhibition in p53 wild-type neuroblastoma. Cell Death Discov 1:15026–. 10.1038/cddiscovery.2015.26 26998348 10.1038/cddiscovery.2015.26 PMC4794278

[20] Sazonova EV, Yapryntseva MA, Pervushin NV et al (2024) Cancer drug resistance: targeting proliferation or programmed cell death. Cells 13:388. 10.3390/cells13050388 38474352 10.3390/cells13050388 PMC10930385

[21] Kocik J, Machula M, Wisniewska A et al (2019) Helping the released guardian: drug combinations for supporting the anticancer activity of HDM2 (MDM2) antagonists. Cancers (Basel) 11:1014. 10.3390/cancers11071014 31331108 10.3390/cancers11071014 PMC6678622

[22] Senichkin VV, Pervushin NV, Zuev AP et al (2020) Targeting Bcl-2 family proteins: what, where, when? Biochemistry (Mosc) 85:1210–1226. 10.1134/S0006297920100090 33202206 10.1134/S0006297920100090

[23] Pervushin NV, Senichkin VV, Zhivotovsky B, Kopeina GS (2020) Mcl-1 as a barrier in cancer treatment: can we target it now? Int Rev Cell Mol Biol 351:23–55. 10.1016/bs.ircmb.2020.01.002 32247581 10.1016/bs.ircmb.2020.01.002

[24] Senichkin VV, Pervushin NV, Zamaraev AV et al (2021) Bak and Bcl-xL participate in regulating sensitivity of solid tumor derived cell lines to Mcl-1 inhibitors. Cancers (Basel) 14:181. 10.3390/cancers14010181 35008345 10.3390/cancers14010181 PMC8750033

[25] Van Goethem A, Yigit N, Moreno-Smith M et al (2017) Dual targeting of MDM2 and BCL2 as a therapeutic strategy in neuroblastoma. Oncotarget 8:57047–57057. 10.18632/oncotarget.18982 28915653 10.18632/oncotarget.18982 PMC5593624

[26] Tolstik E, Gongalsky MB, Dierks J et al (2022) Raman and fluorescence micro-spectroscopy applied for the monitoring of sunitinib-loaded porous silicon nanocontainers in cardiac cells. Front Pharmacol 13:962763. 10.3389/fphar.2022.962763 36016563 10.3389/fphar.2022.962763 PMC9397571

[27] Gongalsky MB, Pervushin NV, Maksutova DE et al (2021) Optical monitoring of the biodegradation of porous and solid silicon nanoparticles. Nanomaterials (Basel) 11:2167. 10.3390/nano11092167 34578485 10.3390/nano11092167 PMC8466475

[28] Pfaffl MW (2001) A new mathematical model for relative quantification in real-time RT-PCR. Nucleic Acids Res 29:e45. 10.1093/nar/29.9.e45 11328886 10.1093/nar/29.9.e45 PMC55695

[29] Wang Y, Rosengarth A, Luecke H (2007) Structure of the human p53 core domain in the absence of DNA. Acta Crystallogr D Biol Crystallogr 63:276–281. 10.1107/S0907444906048499 17327663 10.1107/S0907444906048499

[30] Guex N, Peitsch MC (1997) SWISS-MODEL and the swiss-pdbviewer: an environment for comparative protein modeling. Electrophoresis 18:2714–2723. 10.1002/elps.1150181505 9504803 10.1002/elps.1150181505

[31] Case DA, Belfon K, Ben-Shalom IY, Brozell SR, Cerutti DS, Cheatham TE 3rd, Cruzeiro VWD, Darden TA, Duke RE, Giambasu G, Gilson MK, Gohlke H, Goetz AW, Harris R, Izadi S, Izmailov SA, Kasavajhala K, Kovalenko A, Krasny R, Kurtzman T, Lee TS, LeGrand S, Li P, Lin C, Liu J, Luchko T, Luo R, Man V, Merz KM, Miao Y, Mikhailovskii O, Monard G, Nguyen H, Onufriev A, Pan F, Pantano S, Qi R, Roe DR, Roitberg A, Sagui C, Schott-Verdugo S, Shen J, Simmerling CL, Skrynnikov NR, Smith J, Swails J, Walker RC, Wang J, Wilson L, Wolf RM, Wu X, Xiong Y, Xue Y, York DM, and Kollman PA (2020) AMBER 2020. University of California, San Francisco

[32] Gokcan H, Isayev O (2022) Prediction of protein pK a with representation learning. Chem Sci 13:2462–2474. 10.1039/d1sc05610g 35310485 10.1039/d1sc05610g PMC8864681

[33] Machado MR, Pantano S (2020) Split the charge difference in two! a rule of thumb for adding proper amounts of ions in MD simulations. J Chem Theory Comput 16:1367–1372. 10.1021/acs.jctc.9b00953 31999456 10.1021/acs.jctc.9b00953

[34] Maier JA, Martinez C, Kasavajhala K et al (2015) ff14SB: improving the accuracy of protein side chain and backbone parameters from ff99SB. J Chem Theory Comput 11:3696–3713. 10.1021/acs.jctc.5b00255 26574453 10.1021/acs.jctc.5b00255 PMC4821407

[35] Joung IS, Cheatham TE (2009) Molecular dynamics simulations of the dynamic and energetic properties of alkali and halide ions using water-model-specific ion parameters. J Phys Chem B 113:13279–13290. 10.1021/jp902584c 19757835 10.1021/jp902584c PMC2755304

[36] Li P, Roberts BP, Chakravorty DK, Merz KM (2013) Rational design of particle mesh ewald compatible Lennard-Jones parameters for + 2 metal cations in explicit solvent. J Chem Theory Comput 9:2733–2748. 10.1021/ct400146w 23914143 10.1021/ct400146w PMC3728907

[37] Salomon-Ferrer R, Götz AW, Poole D et al (2013) Routine microsecond molecular dynamics simulations with AMBER on GPUs. 2. Explicit solvent particle mesh ewald. J Chem Theory Comput 9:3878–3888. 10.1021/ct400314y 26592383 10.1021/ct400314y

[38] Le Grand S, Götz AW, Walker RC (2013) SPFP: speed without compromise—a mixed precision model for GPU accelerated molecular dynamics simulations. Comput Phys Commun 184:374–380. 10.1016/j.cpc.2012.09.022

[39] Nilov DK, Zamaraev AV, Zhivotovsky B, Kopeina GS (2022) Exploring caspase mutations and post-translational modification by molecular modeling approaches. J Vis Exp. 10.3791/64206 36314804 10.3791/64206

[40] Roe DR, Cheatham TE (2013) PTRAJ and CPPTRAJ: software for processing and analysis of molecular dynamics trajectory data. J Chem Theory Comput 9:3084–3095. 10.1021/ct400341p 26583988 10.1021/ct400341p

[41] Humphrey W, Dalke A, Schulten K (1996) VMD: visual molecular dynamics. J Mol Graph 14:33–38. 10.1016/0263-7855(96)00018-5 8744570 10.1016/0263-7855(96)00018-5

[42] Plesca D, Mazumder S, Almasan A (2008) DNA damage response and apoptosis. Methods Enzymol 446:107–122. 10.1016/S0076-6879(08)01606-6 18603118 10.1016/S0076-6879(08)01606-6 PMC2911482

[43] Lazebnik YA, Kaufmann SH, Desnoyers S et al (1994) Cleavage of poly(ADP-ribose) polymerase by a proteinase with properties like ICE. Nature 371:346–347. 10.1038/371346a0 8090205 10.1038/371346a0

[44] Crowley LC, Marfell BJ, Scott AP et al (2016) Dead cert: measuring cell death. Cold Spring Harb Protoc. 10.1101/pdb.top070318 27934691 10.1101/pdb.top070318

[45] Ahmed Juvale II, Abdul Hamid AA, Abd Halim KB, Che Has AT (2022) P-glycoprotein: new insights into structure, physiological function, regulation and alterations in disease. Heliyon 8:e09777. 10.1016/j.heliyon.2022.e09777 35789865 10.1016/j.heliyon.2022.e09777 PMC9249865

[46] Michaelis M, Rothweiler F, Klassert D et al (2009) Reversal of P-glycoprotein-mediated multidrug resistance by the murine double minute 2 antagonist nutlin-3. Cancer Res 69:416–421. 10.1158/0008-5472.CAN-08-1856 19147553 10.1158/0008-5472.CAN-08-1856

[47] Holstege H, Joosse SA, van Oostrom CTM et al (2009) High incidence of protein-truncating TP53 mutations in BRCA1-related breast cancer. Cancer Res 69:3625–3633. 10.1158/0008-5472.CAN-08-3426 19336573 10.1158/0008-5472.CAN-08-3426

[48] Tian X, Dai S, Sun J et al (2017) The association between the TP53 Arg72Pro polymorphism and colorectal cancer: an updated meta-analysis based on 32 studies. Oncotarget 8:1156–1165. 10.18632/oncotarget.13589 27901479 10.18632/oncotarget.13589 PMC5352043

[49] Klug SJ, Ressing M, Koenig J et al (2009) TP53 codon 72 polymorphism and cervical cancer: a pooled analysis of individual data from 49 studies. Lancet Oncol 10:772–784. 10.1016/S1470-2045(09)70187-1 19625214 10.1016/S1470-2045(09)70187-1

[50] Soussi T (2022) Benign SNPs in the coding region of TP53: finding the needles in a haystack of pathogenic variants. Cancer Res 82:3420–3431. 10.1158/0008-5472.CAN-22-0172 35802772 10.1158/0008-5472.CAN-22-0172

[51] Marine J-CW, Dyer MA, Jochemsen AG (2007) MDMX: from bench to bedside. J Cell Sci 120:371–378. 10.1242/jcs.03362 17251377 10.1242/jcs.03362

[52] Tanaka T, Watanabe M, Yamashita K (2018) Potential therapeutic targets of TP53 gene in the context of its classically canonical functions and its latest non-canonical functions in human cancer. Oncotarget 9:16234–16247. 10.18632/oncotarget.24611 29662640 10.18632/oncotarget.24611 PMC5882331

[53] Timofeev O, Stiewe T (2021) Rely on each other: DNA binding cooperativity shapes p53 functions in tumor suppression and cancer therapy. Cancers (Basel) 13:2422. 10.3390/cancers13102422 34067731 10.3390/cancers13102422 PMC8155944

[54] Miao Y, Feher VA, McCammon JA (2015) Gaussian accelerated molecular dynamics: unconstrained enhanced sampling and free energy calculation. J Chem Theory Comput 11:3584–3595. 10.1021/acs.jctc.5b00436 26300708 10.1021/acs.jctc.5b00436 PMC4535365

[55] Wang J, Arantes PR, Bhattarai A et al (2021) Gaussian accelerated molecular dynamics (GaMD): principles and applications. Wiley Interdiscip Rev Comput Mol Sci 11:e1521. 10.1002/wcms.1521 34899998 10.1002/wcms.1521 PMC8658739

[56] Lukman S, Lane DP, Verma CS (2013) Mapping the structural and dynamical features of multiple p53 DNA binding domains: insights into loop 1 intrinsic dynamics. PLoS ONE 8:e80221. 10.1371/journal.pone.0080221 24324553 10.1371/journal.pone.0080221 PMC3855832

[57] Cho Y, Gorina S, Jeffrey PD, Pavletich NP (1994) Crystal structure of a p53 tumor suppressor-DNA complex: understanding tumorigenic mutations. Science 265:346–355. 10.1126/science.8023157 8023157 10.1126/science.8023157

[58] Weinberg RL, Veprintsev DB, Fersht AR (2004) Cooperative binding of tetrameric p53 to DNA. J Mol Biol 341:1145–1159. 10.1016/j.jmb.2004.06.071 15321712 10.1016/j.jmb.2004.06.071

[59] Gaglia G, Guan Y, Shah JV, Lahav G (2013) Activation and control of p53 tetramerization in individual living cells. Proc Natl Acad Sci U S A 110:15497–15501. 10.1073/pnas.1311126110 24006363 10.1073/pnas.1311126110 PMC3780836

[60] McLure KG, Lee PW (1998) How p53 binds DNA as a tetramer. EMBO J 17:3342–3350. 10.1093/emboj/17.12.3342 9628871 10.1093/emboj/17.12.3342 PMC1170672

[61] Monti P, Perfumo C, Bisio A et al (2011) Dominant-negative features of mutant TP53 in germline carriers have limited impact on cancer outcomes. Mol Cancer Res 9:271–279. 10.1158/1541-7786.MCR-10-0496 21343334 10.1158/1541-7786.MCR-10-0496 PMC3077904

[62] Di Como CJ, Prives C (1998) Human tumor-derived p53 proteins exhibit binding site selectivity and temperature sensitivity for transactivation in a yeast-based assay. Oncogene 16:2527–2539. 10.1038/sj.onc.1202041 9627118 10.1038/sj.onc.1202041

[63] Shi Z, Moult J (2011) Structural and functional impact of cancer-related missense somatic mutations. J Mol Biol 413:495–512. 10.1016/j.jmb.2011.06.046 21763698 10.1016/j.jmb.2011.06.046 PMC4177034

[64] Aziz MH, Shen H, Maki CG (2011) Acquisition of p53 mutations in response to the non-genotoxic p53 activator Nutlin-3. Oncogene 30:4678–4686. 10.1038/onc.2011.185 21643018 10.1038/onc.2011.185 PMC3347888

[65] Michaelis M, Rothweiler F, Barth S et al (2011) Adaptation of cancer cells from different entities to the MDM2 inhibitor nutlin-3 results in the emergence of p53-mutated multi-drug-resistant cancer cells. Cell Death Dis 2:e243. 10.1038/cddis.2011.129 22170099 10.1038/cddis.2011.129 PMC3252738

[66] Berberich A, Kessler T, Thomé CM et al (2019) Targeting resistance against the MDM2 inhibitor RG7388 in glioblastoma cells by the MEK inhibitor trametinib. Clin Cancer Res 25:253–265. 10.1158/1078-0432.CCR-18-1580 30274984 10.1158/1078-0432.CCR-18-1580

[67] Skalniak L, Kocik J, Polak J et al (2018) Prolonged Idasanutlin (RG7388) treatment leads to the generation of p53-mutated cells. Cancers (Basel) 10:396. 10.3390/cancers10110396 30352966 10.3390/cancers10110396 PMC6266412

[68] de Andrade KC, Lee EE, Tookmanian EM et al (2022) The TP53 database: transition from the international agency for research on cancer to the US national cancer institute. Cell Death Differ 29:1071–1073. 10.1038/s41418-022-00976-3 35352025 10.1038/s41418-022-00976-3 PMC9090805

[69] AACR Project GENIE Consortium (2017) AACR project GENIE: powering precision medicine through an international consortium. Cancer Discov 7:818–831. 10.1158/2159-8290.CD-17-0151 28572459 10.1158/2159-8290.CD-17-0151 PMC5611790

[70] Colman MS, Afshari CA, Barrett JC (2000) Regulation of p53 stability and activity in response to genotoxic stress. Mutat Res 462:179–188. 10.1016/s1383-5742(00)00035-1 10767629 10.1016/s1383-5742(00)00035-1

[71] Tran HC, Marachelian A, Venkatramani R et al (2015) Oxaliplatin and doxorubicin for relapsed or refractory high-risk neuroblastoma. Pediatr Hematol Oncol 32:26–31. 10.3109/08880018.2014.983624 25551355 10.3109/08880018.2014.983624

[72] Van Maerken T, Rihani A, Dreidax D et al (2011) Functional analysis of the p53 pathway in neuroblastoma cells using the small-molecule MDM2 antagonist nutlin-3. Mol Cancer Ther 10:983–993. 10.1158/1535-7163.MCT-10-1090 21460101 10.1158/1535-7163.MCT-10-1090

[73] Corvi R, Savelyeva L, Breit S et al (1995) Non-syntenic amplification of MDM2 and MYCN in human neuroblastoma. Oncogene 10:1081–1086 7700632

[74] Cattelani S, Defferrari R, Marsilio S et al (2008) Impact of a single nucleotide polymorphism in the MDM2 gene on neuroblastoma development and aggressiveness: results of a pilot study on 239 patients. Clin Cancer Res 14:3248–3253. 10.1158/1078-0432.CCR-07-4725 18519749 10.1158/1078-0432.CCR-07-4725

[75] Rayburn E, Zhang R, He J, Wang H (2005) MDM2 and human malignancies: expression, clinical pathology, prognostic markers, and implications for chemotherapy. Curr Cancer Drug Targets 5:27–41. 10.2174/1568009053332636 15720187 10.2174/1568009053332636

[76] Alvarado-Ortiz E, de la Cruz-López KG, Becerril-Rico J et al (2020) Mutant p53 gain-of-function: role in cancer development, progression, and therapeutic approaches. Front Cell Dev Biol 8:607670. 10.3389/fcell.2020.607670 33644030 10.3389/fcell.2020.607670 PMC7905058

[77] Pitolli C, Wang Y, Mancini M et al (2019) Do mutations turn p53 into an oncogene? Int J Mol Sci 20:6241. 10.3390/ijms20246241 31835684 10.3390/ijms20246241 PMC6940991

[78] Andreeff M, Kelly KR, Yee K et al (2016) Results of the phase I trial of RG7112, a small-molecule MDM2 antagonist in leukemia. Clin Cancer Res 22:868–876. 10.1158/1078-0432.CCR-15-0481 26459177 10.1158/1078-0432.CCR-15-0481 PMC4809642

[79] Montesinos P, Beckermann BM, Catalani O et al (2020) MIRROS: a randomized, placebo-controlled, phase III trial of cytarabine ± idasanutlin in relapsed or refractory acute myeloid leukemia. Future Oncol 16:807–815. 10.2217/fon-2020-0044 32167393 10.2217/fon-2020-0044

[80] Deben C, Boullosa LF, Domen A et al (2021) Characterization of acquired nutlin-3 resistant non-small cell lung cancer cells. Cancer Drug Resist 4:233–243. 10.20517/cdr.2020.91 35582010 10.20517/cdr.2020.91 PMC9019186

[81] Li L, Zhang Y, Gao Y et al (2023) LncSNHG14 promotes nutlin3a resistance by inhibiting ferroptosis via the miR-206 /SLC7A11 axis in osteosarcoma cells. Cancer Gene Ther 30:704–715. 10.1038/s41417-022-00581-z 36599973 10.1038/s41417-022-00581-z

