# Supplementary material for: BH3-mimetics or DNA-damaging agents in combination with RG7388 overcome p53 mutation-induced resistance to MDM2 inhibition
Source: Apoptosis. 2024 Sep 2;29(11-12):2197–213. doi: 10.1007/s10495-024-02014-8 (PMC11550243; doi:10.1007/s10495-024-02014-8)
Supplement: Supplementary file 1 — Supplementary material 1 (DOCX 1603 kb) [file 10495_2024_2014_MOESM1_ESM.docx]

**Supplementary Figures**

**
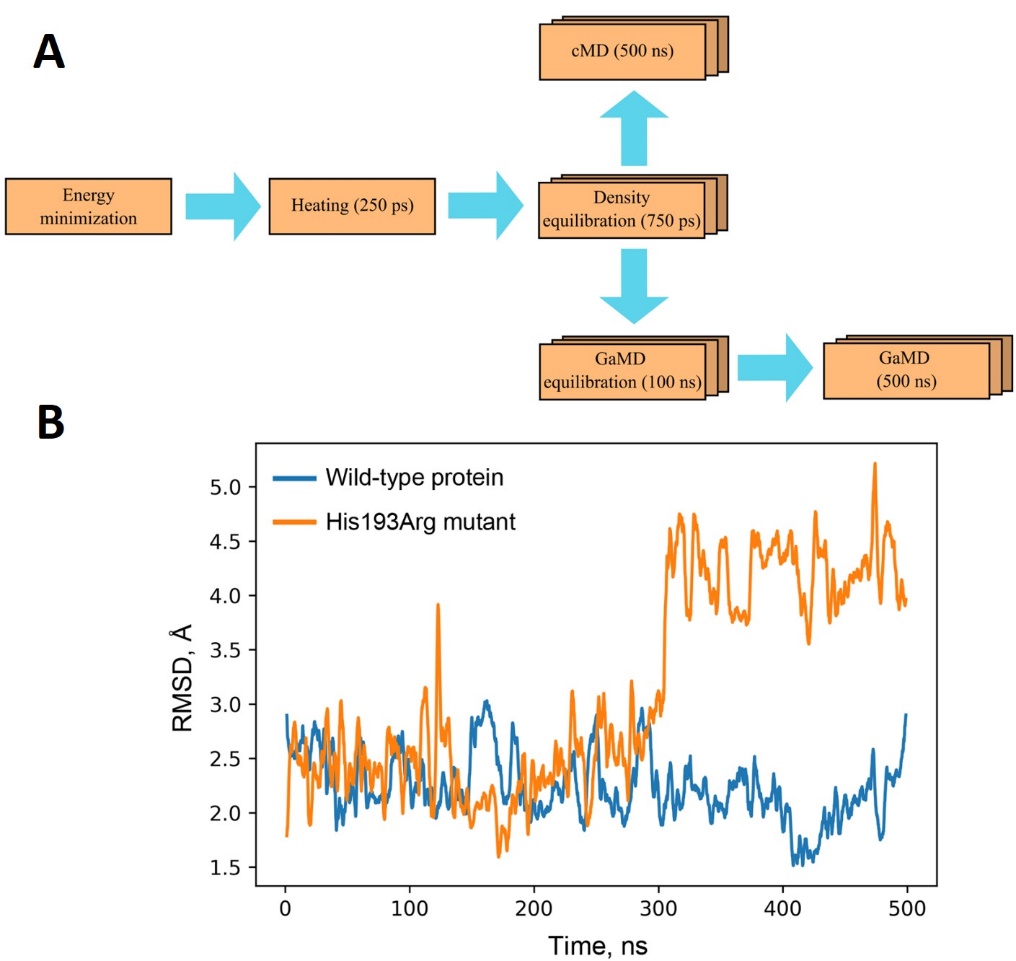
**

**Figure S1. Schematic representation of p53 simulations (A) and root-mean-square deviation (RMSD) of the loop 182–194 from its initial position (obtained at the energy minimization stage) during GaMD simulations of p53 (B).** Trajectory frames were superimposed onto the starting structure by fitting the backbone atoms of the protein, and then RMSD of the loop 182–194 backbone was calculated. A major conformational change in the His193Arg mutant structure was observed at *t* ≈ 300 ns.

**
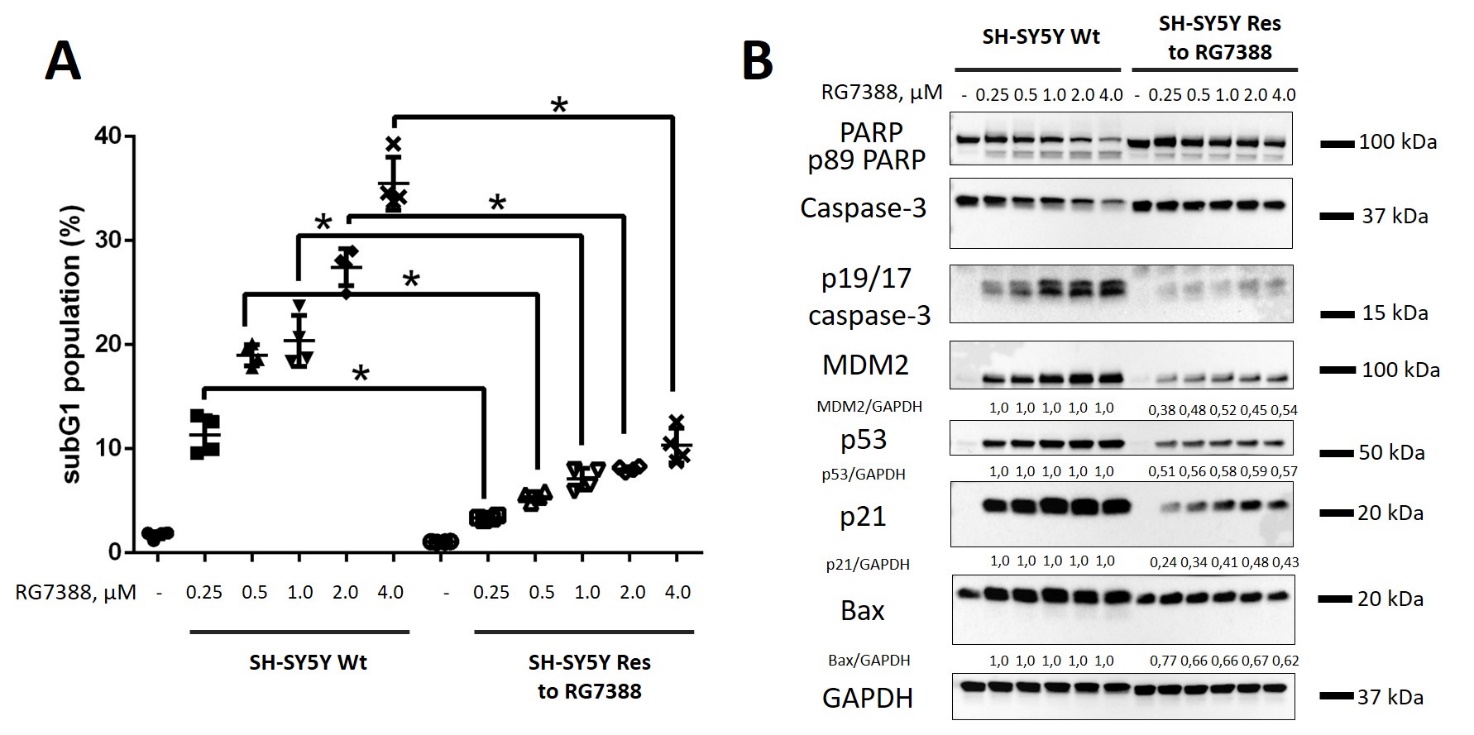
**

**Figure S2. Analysis of the efficiency of RG7388 in wild-type and RG7388-resistant neuroblastoma cells (SH-SY5Y Wt and SH-SY5Y Res, respectively).** SubG1 test (**A**) and WB analysis (**B**) of SH-SY5Y (parental and resistant cells) cells upon treatment with RG7388 at 0.25 – 4 µM. Densitometric analysis of p53, p21, Bax, and MDM2 levels normalized to GAPDH. Results are presented as mean ± standard deviation (SD), n = 4 (Mann-Whitney U-test), p < 0.05, n.s. — not significant. GAPDH was used as a loading control. Incubation time: 24 h.

**
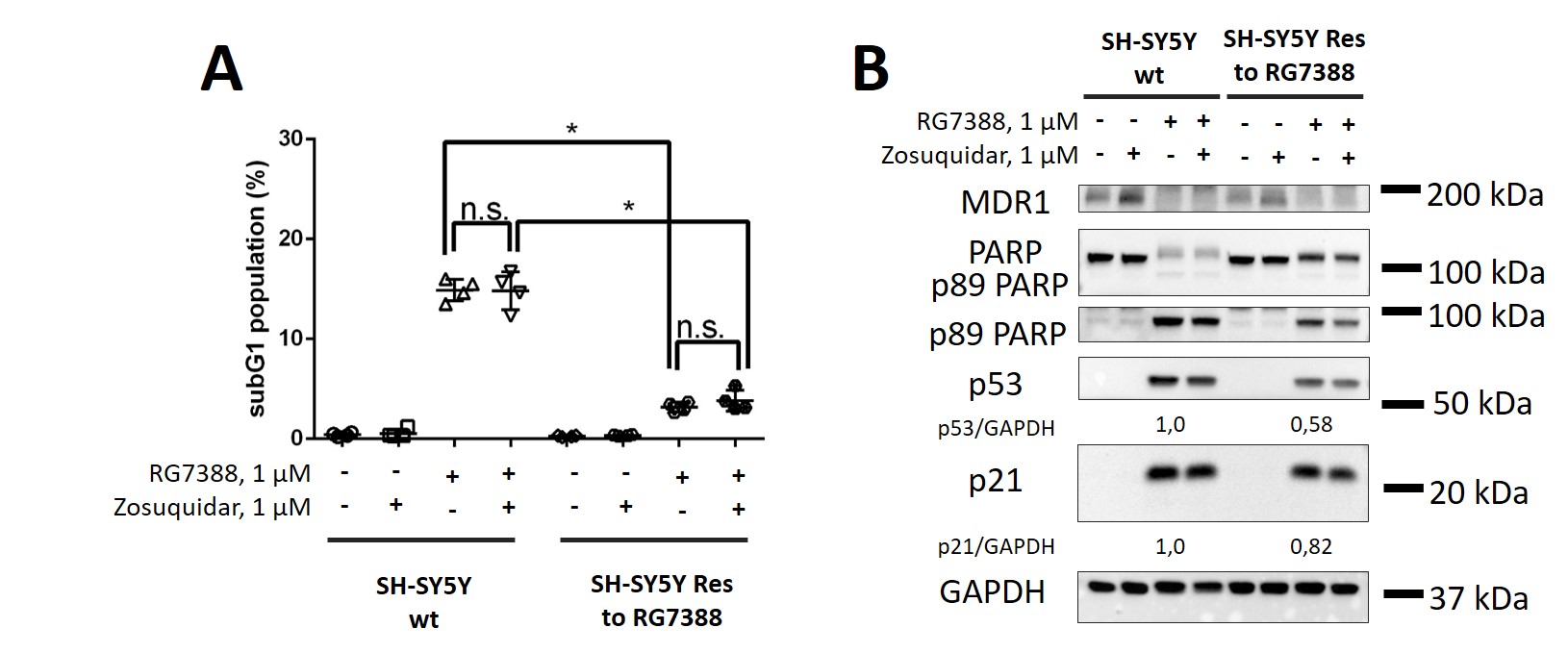
**

**Figure S3. Analysis of the efficiency of the combination RG7388 and an MDR1 inhibitor, Zosuquidar, in wild-type and RG7388-resistant neuroblastoma cells (SH-SY5Y Wt and SH-SY5Y Res, respectively).** SubG1 test (**A**) and WB analysis (**B**) of SH-SY5Y Wt and SH-SY5Y Res to RG7388 cells upon treatment with RG7388 and Zosuquidar (both – 1 µM, 24 h). Densitometric analysis of p53 and p21 levels normalized to GAPDH. Results are presented as mean ± standard deviation (SD), n = 4 (Mann-Whitney U-test), p < 0.05, n.s. — not significant. GAPDH was used as a loading control.

**
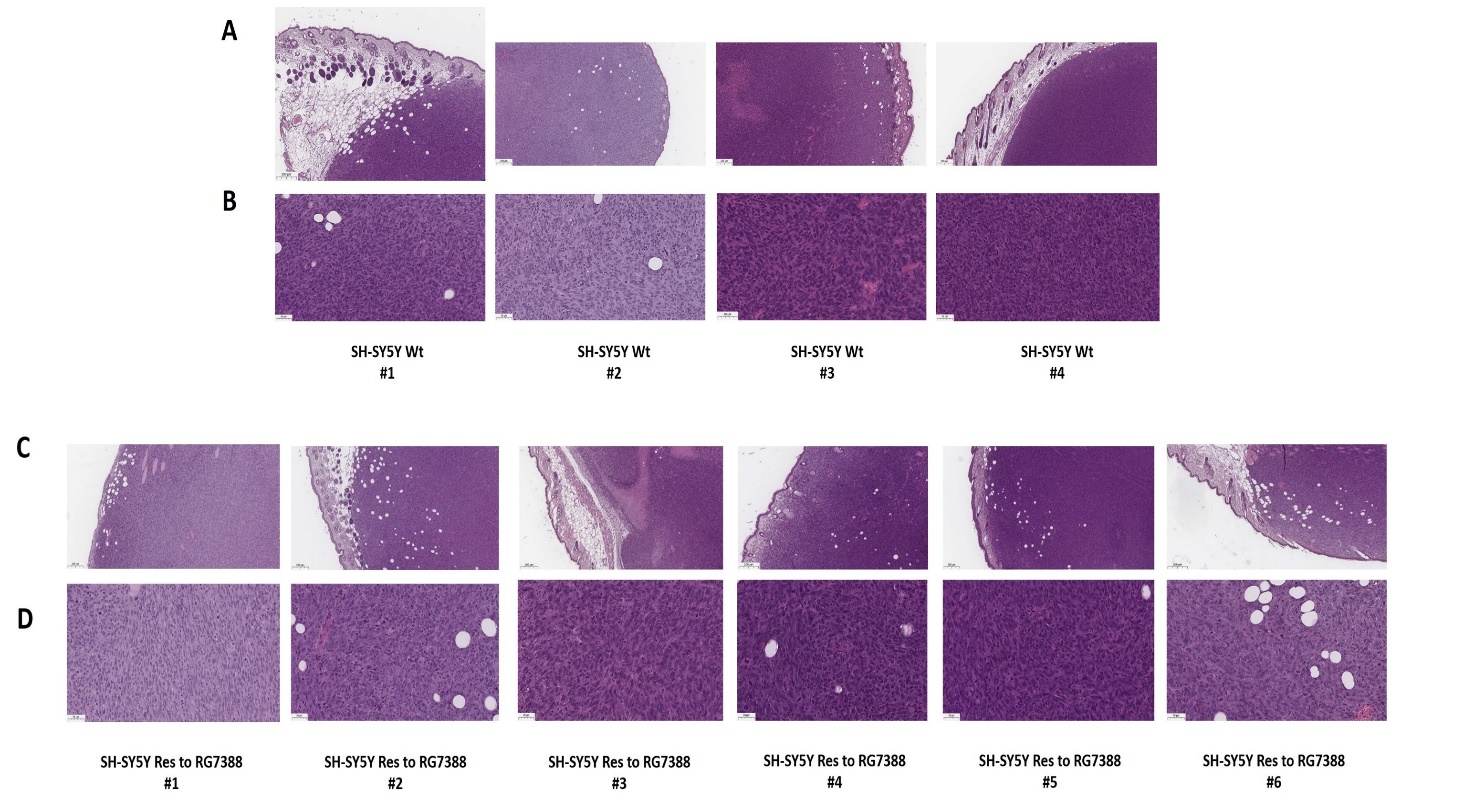
**

**Figure S4. Histological analysis of tumors bearing wild-type (A, B) (n = 4) and resistant to RG7388 SH-SY5Y cells (C, D) (n = 6) obtained from mouse xenografts after hematoxylin and eosin staining.** The representative images of tumor tissues.


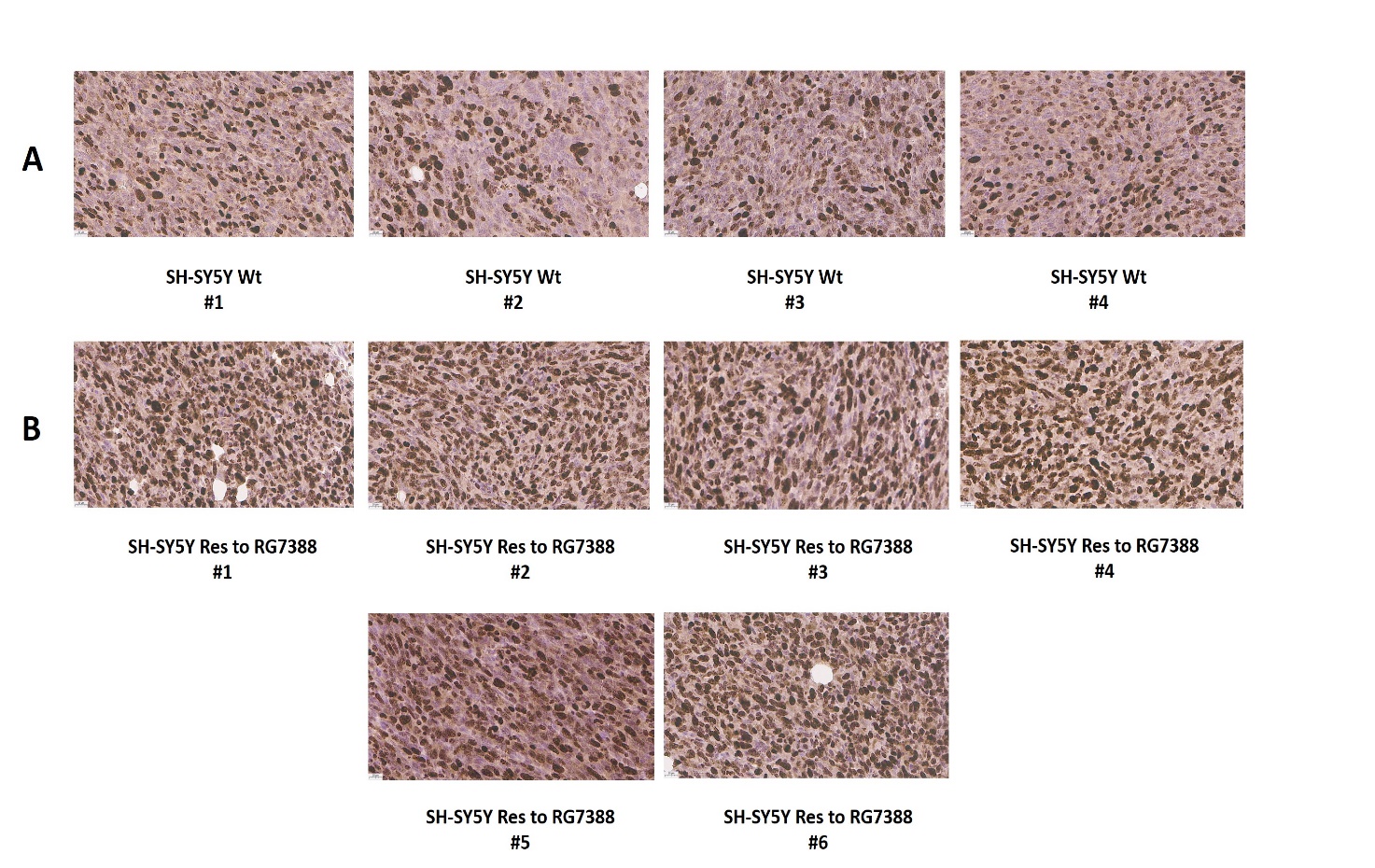


**Figure S5. Ki67 immunohistochemical staining of tumors bearing Wt (A) (n = 4) and resistant to RG7388 SH-SY5Y cells (B) (n = 6) obtained from mouse xenografts.** The representative images of tumor tissues.


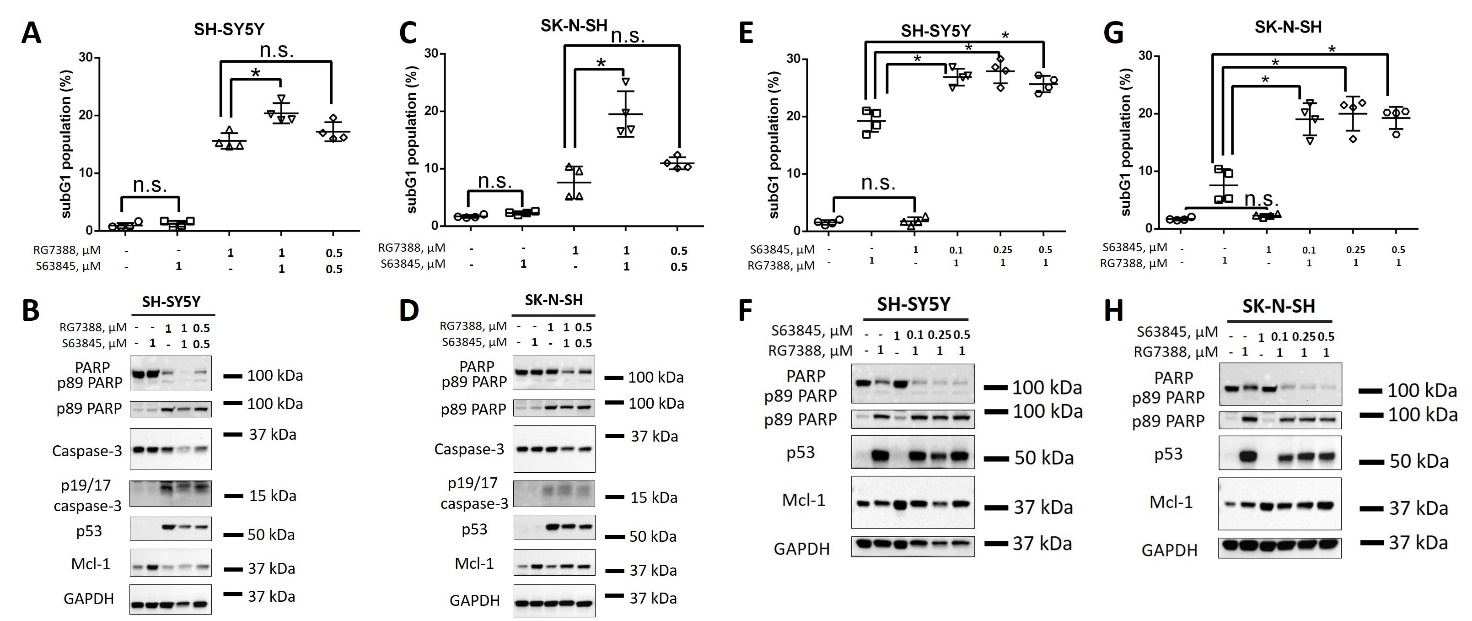


**Figure S6. Analysis of the efficiency of simultaneous inhibition of Mcl-2 (S63845) and MDM2 (RG7388) in SH-SY5Y and SK-N-SH cells.** subG1 test (**A, C, E, G**) and WB analysis (**B, D, F, H**) of SH-SY5Y (**A, B**) and SK-N-SH (**C, D**) cells upon treatment with S63845 and RG7388 at indicated concentrations. Results are presented as mean ± standard deviation (SD), n=4 (Mann-Whitney U-test), * p < 0.05, n.s. — not significant. GAPDH was used as a loading control. Time of incubation: 24 h.

**Supplementary Tables**

**Table S1. List of primers used in RT-qPCR analysis.**

| **Gene** | **Forward primer** | **Reverse primer** |
| --- | --- | --- |
| *MDM2* | GCAGTGAATCTACAGGGACG | TCCTGATCCAACCAATCACCT |
| *CDKN1A* | TGGAGACTCTCAGGGTCGAAA | GGCGTTTGGAGTGGTAGAAATC |
| *BAX* | GTCGCCCTTTTCTACTTTGC | CGGAGGAAGTCCAATGTCC |
| *BBC3* | CCTCAACGCACAGTACGAG | GCACCTAATTGGGCTCCATC |
| *TBP* | GAGAGTTCTGGGATTGTACC | GGATTATATTCGGCGTTTCG |

**Table S2. Control data used for p53 simulations in Amber.**

| **Energy minimization, stage 1** |
| --- |
| &cntrl  imin=1, maxcyc=5000, ncyc=2500,  cut=10.0, ntb=1,  ntc=1, ntf=1,  ntpr=100,  ntr=1,  restraintmask=':1-197&!@H=',  restraint_wt=2.0  / |
| **Energy minimization, stage 2** |
| &cntrl  imin=1, maxcyc=10000, ncyc=5000,  cut=10.0, ntb=1,  ntc=1, ntf=1,  ntpr=100  / |
| **Heating** |
| &cntrl  irest=0, ntx=1,  nstlim=125000, dt=0.002,  ntpr=1000, ntwx=1000,  ntc=2, ntf=2,  cut=10.0, ntb=1,  ntt=3, gamma_ln=2.0,  tempi=0.0, temp0=300.0,  ntr=1, restraintmask=':1-197&!@H=',  restraint_wt=1.0,  nmropt=1  /  &wt TYPE='TEMP0',  istep1=0, istep2=125000,  value1=0.1, value2=300.0 /  &wt TYPE='END' / |
| **Density equilibration** |
| &cntrl  irest=1, ntx=5,  nstlim=375000, dt=0.002,  ntpr=1000, ntwx=1000,  ntc=2, ntf=2,  cut=10.0, ntb=2,  ntt=3, gamma_ln=2.0, temp0=300.0,  ntp=1, taup=2.0, barostat=1  / |
| **cMD production** |
| &cntrl  irest=1, ntx=5,  nstlim=250000000, dt=0.002,  ntpr=1000, ntwx=1000, ntwr=2500000,  ntc=2, ntf=2,  cut=10.0, ntb=2,  ntt=3, gamma_ln=2.0, temp0=300.0,  ntp=1, taup=2.0, barostat=2  / |
| **GaMD equilibration^*^** |
| &cntrl  irest=1, ntx=5,  nstlim=50000000, dt=0.002,  ntpr=1000, ntwx=1000, ntwr=2500000,  ntc=2, ntf=2,  cut=10.0, ntb=2,  ntt=3, gamma_ln=2.0, temp0=300.0,  ntp=1, taup=2.0, barostat=2,  igamd=3, iE=1, irest_gamd=0,  ntcmdprep=0, ntcmd=1000000,  ntebprep=4000000, nteb=45000000,  ntave=200000,  sigma0D=6.0, sigma0P=6.0  / |
| **GaMD production** |
| &cntrl  irest=1, ntx=5,  nstlim=250000000, dt=0.002,  ntpr=1000, ntwx=1000, ntwr=2500000,  ntc=2, ntf=2,  cut=10.0, ntb=2,  ntt=3, gamma_ln=2.0, temp0=300.0,  ntp=1, taup=2.0, barostat=2,  igamd=3, iE=1, irest_gamd=1,  ntcmdprep=0, ntcmd=0,  ntebprep=0, nteb=0,  ntave=200000,  sigma0D=6.0, sigma0P=6.0  / |

^*^ In GaMD simulations the dual potential boost (on both dihedral and total potential energy) was applied with default σ_0P_ and σ_0D_ values (6 kcal/mol), and threshold energy E was set to the lower bound. GaMD equilibration included ntcmd steps (2 ns) followed by ntebprep steps (8 ns) and nteb steps (90 ns); potential energy statistics were updated every 200,000 simulation steps.

**Table S3. Values of minimum, average, maximum, and standard deviation of the potential energy (kcal/mol) were used to calculate the boost potentials when running GaMD production simulations of p53.**

| **Wt protein** | | | | |
| --- | --- | --- | --- | --- |
| **Potential energy statistics** | **V_min_** | **V_avg_** | **V_max_** | **σ_V_** |
| Dihedral potential energy | 2386 | 2633 | 2696 | 12.09 |
| Total potential energy | –113495 | –109811 | –109579 | 44.91 |
| **His193Arg mutant** | | | | |
| **Potential energy statistics** | **V_min_** | **V_avg_** | **V_max_** | **σ_V_** |
| Dihedral potential energy | 2386 | 2631 | 2692 | 11.83 |
| Total potential energy | –113770 | –110064 | –109835 | 45.22 |

**Table S4. Histological analysis of tumors bearing wild-type (n = 4) and resistant SH-SY5Y cells (n = 6) obtained from mouse xenografts.**

|  | **Tumor sample** | **Maximum size of tumor (mm)** | **Mitotic index (10 HPF ×400)** | **Apoptotic index (10 HPF ×400)** | **M/A ratio** | **Ki-67 (%)** |
| --- | --- | --- | --- | --- | --- | --- |
| **SH-SY5Y Wt** | 1 | 12 | 31 | 21 | 1,48 | 50 |
|  | 2 | 12 | 30 | 18 | 1,67 | 40 |
|  | 3 | 12 | 24 | 11 | 2,18 | 45 |
|  | 4 | 8 | 20 | 9 | 2,22 | 50 |
|  | **Total (mean + SD)** | **11 ± 2** | **26,25 ± 5,19** | **14,75 ± 5,68** | **1,89 ± 0,37** | **46,25 ± 4,79** |
| **SH-SY5Y Res to RG7388** | 1 | 11 | 38 | 9 | 4,22 | 70 |
|  | 2 | 9 | 21 | 12 | 1,75 | 75 |
|  | 3 | 10 | 38 | 11 | 3,45 | 85 |
|  | 4 | 8 | 40 | 13 | 3,08 | 85 |
|  | 5 | 9 | 36 | 15 | 2,40 | 70 |
|  | 6 | 14 | 39 | 10 | 3,90 | 90 |
|  | **Total (mean + SD)** | **10,17 ± 2,14** | **35,33 ± 7,15** | **11,67 ± 2,16** | **3,13 ± 0,93** | **79,17 ± 8,61** |
|  | Mann-Whitney U-test | n.s. (p=0.5) | n.s. (p=0.057) | n.s. (p=0.58) | * (p=0.038) | * (p=0.0095) |

Results are presented as mean ± standard deviation (SD), Mann-Whitney U-test, * p < 0.05, n.s. — not significant.
